# Sustainable tandem acylation/Diels–Alder reaction toward versatile tricyclic epoxyisoindole-7-carboxylic acids in renewable green solvents

**DOI:** 10.3762/bjoc.20.114

**Published:** 2024-06-06

**Authors:** Ayhan Yıldırım

**Affiliations:** 1 Department of Chemistry, Bursa Uludağ University, Bursa 16059, Turkey

**Keywords:** biobased solvent, epoxyisoindoles, furanics, green chemistry, intramolecular Diels–Alder reaction

## Abstract

Tandem Diels–Alder reactions are often used for the straightforward formation of complex natural compounds and the fused polycyclic systems contained in their precursors. In the second step of this reaction, regio- and stereochemically controlled intramolecular cyclization leads to the formation of versatile nitrogen-containing tricyclic systems. However, these useful organic transformations are usually carried out in highly toxic organic solvents such as benzene, toluene, chloroform, etc. Despite recent efforts by 'green chemists', synthetic chemists still use these traditional toxic organic solvents in many of their reactions, even though safer alternatives are available. However, in addition to the harmful effects of these petrochemical solvents on the environment, the prediction that their resources will run out in the near future has led 'green chemists' to explore solvents that can be derived from renewable resources and used effectively in various organic transformations. In this context, we have shown for the first time that the 100% atom-economical tandem Diels–Alder reaction between aminofuranes and maleic anhydride can be carried out successfully in vegetable oils and waxes. The reaction was successfully carried out in sunflower seed oil, olive oil, oleic acid and lauryl myristate under mild reaction conditions. A series of epoxyisoindole-7-carboxylic acid and bisepoxyisoindole-7-carboxylic acids were obtained in good yields after a practical isolation procedure. The results obtained in this study demonstrate the potential of vegetable oils and their renewable materials to provide a reaction medium that is more sustainable than conventional organic solvents in cascade Diels–Alder reactions and can be used repeatedly without significant degradation. These materials also allow the reaction to be completed in less time, with less energy consumption and higher yields.

## Introduction

For many years, fossil fuels have provided the chemicals needed by many industries, particularly the chemical and pharmaceutical industries [1]. It is well known that the processing of these resources and the steps involved in converting them into various chemical compounds are among the main sources of serious environmental problems [2]. However, the risk of depletion of these resources in the near future has led researchers to research and develop safer, environmentally friendly and renewable resources [3–5]. Biomass is an important and more economical renewable source of hydrocarbons and a variety of intermediates that are needed by a wide range of industries [6–14].

Worldwide, vegetable oils are mainly produced for food and feed purposes, but a small number of specific oils are also produced as a source of materials for industrial applications [15–16]. These oils are of increasing interest for the production of a wide range of polymeric materials [17–23], drug delivery systems [24–29], less toxic anticancer drugs [30–33] and intermediates suitable for various organic transformations [14,34–38]. In addition to their unique properties, vegetable oils are known to be useful as green solvents in many applications [39–42]. The evaluation of vegetable oils as alternative solvents in organic synthesis is very limited [43–47].

Synthetic chemists are still trying to make the conditions for Diels–Alder reactions more environmentally friendly [48–58]. However, the number and variety of substrates in these studies is often limited, deep eutectic solvents are required and it is difficult to achieve both satisfactory product yields and the desired stereoselectivity [59–61]. The viscous nature of the aminofuranes usually requires the use of toxic solvents such as toluene, and in some studies, solvents such as DMSO, which are difficult to remove from the reaction medium [62–64]. Although solvent effects are of little importance in intramolecular Diels–Alder reactions, the effects of solvents such as glycerol, polyethylene glycol, organic carbonates, deep eutectic solvents, supercritical CO_2_ and H_2_O have recently been extensively studied [65–67]. Recently, attention has also been drawn to photoinduced oxidative [4 + 2] annulation reactions [68–70].

This study was the first to evaluate the performances of sunflower seed oil (SSO), olive oil (OO), oleic acid (OA) and lauryl myristate (LM) as green solvents in the well-known intramolecular Diels–Alder furan (IMDAF) reaction between aminofuranes and maleic anhydride. This is a useful approach for the total synthesis of complex natural compounds, epoxyisoindole skeleton-based precursors for various polycyclic compounds, and bio-based homo- and copolymers [71–98]. Therefore, in the present study, we report for the first time a milder, greener strategy to prepare a series of epoxyisoindolinones from aminofuranes and maleic anhydride via cascade amidation and stereocontrolled IMDAF reaction in bio-based solvents as an alternative reaction medium.

## Results and Discussion

In this study, the course of the reaction was first investigated in aqueous micellar media using cetyltrimethylammonium bromide (CTAB), a cationic surfactant. The aminofuran and maleic anhydride shown in Table 1 were reacted with 10% CTAB in the same molar ratios. However, contrary to what some previous studies have reported, hydrophobic packing did not accelerate the reaction and there was no transformation in the reaction within 10 h at room temperature [99–106]. This result suggests that it would be difficult to carry out the relevant IMDAF reactions in water due to the remarkable lack of water solubility of the highly hydrophobic aminofurans selected in this study. Therefore, the decision was made to perform these reactions using vegetable oil as an alternative green solvent system. For this purpose, SSO, OO, OA and LM, which can act as potential solvents, were evaluated to determine the optimum conditions for the reaction (Table 1). Some physical parameters of these materials used as reaction media are listed in Table S1 (Supporting Information File 1). The lauryl myristate used as reaction medium in this study was synthesized in our laboratory according to a previously published procedure [107].

**Table 1:** Optimization of IMDAF cycloaddition using different solvents.^a^

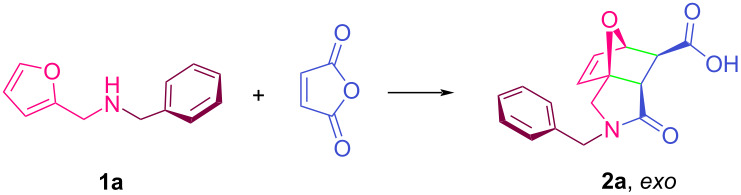				

Entry	Solvent	Temperature (°C)	Time (h)	Yield (%)^b^

1	SSO	rt	3	89
2	SSO	rt	6	91
3	SSO	rt	15	90
4	SSO	50	0.5	96
5	SSO	50	1	91
6	OO	50	0.5	96
7	OA	50	0.5	99
8	benzene	rt	48	89
9	LM	50	0.5	99

^a^Reaction conditions: *N*-benzyl-1-(furan-2-yl)methanamine (**1a**, 1.07 mmol), maleic anhydride (1.07 mmol), solvent: SSO, OO or OA (2 mL) or LM (1 g). ^b^Isolated yields.

The starting aminofuranes were easily prepared by reduction of Schiff bases (formed by condensation of the corresponding mono- or bis-amines with furfural) with sodium borohydride in methanol [83,108–111]. In order to determine the optimum reaction conditions, a series of reactions between *N*-benzyl-1-(furan-2-yl)methanamine (**1a**) and maleic anhydride were carried out and the results obtained are shown in Table 1. As can be seen from the table, SSO, OO, OA and LM are all excellent solvents for the IMDAF reaction and allow the formation of the corresponding addition product in good yields in a very short time and under mild reaction conditions. In particular, in OA and LM, the corresponding reaction takes place in quantitative terms (Table 1, entries 7 and 9). SSO is known to be rich in unsaturated fatty acids, can remain in liquid form over a wide temperature range, is economical and is at the forefront of production and use in various sectors worldwide [112]. In addition, despite its high unsaturation content, SSO has very good thermal stability compared to OO, even at temperatures above 150 °C under normal atmospheric conditions [113]. Both diene and dienophile systems are readily soluble in the reaction medium of interest under the conditions studied, in contrast to aqueous media. The non-polar aprotic nature of vegetable oils and wax esters makes them inert to many reagents, as in this study. The reaction was completed in a much shorter time (Table 1, entry 4) when the temperature of the reaction medium was increased to 50 °C.

**Figure 1:**
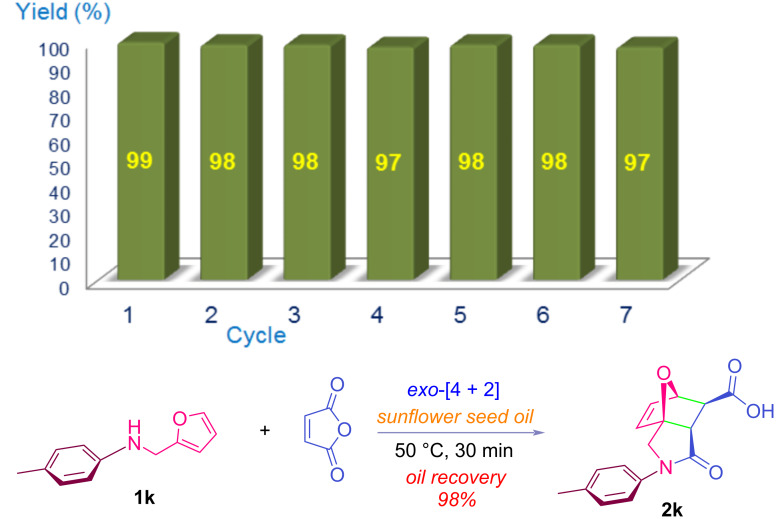
Reaction yields after seven uses of SSO and average recovery of the oil.

**Scheme 1:**
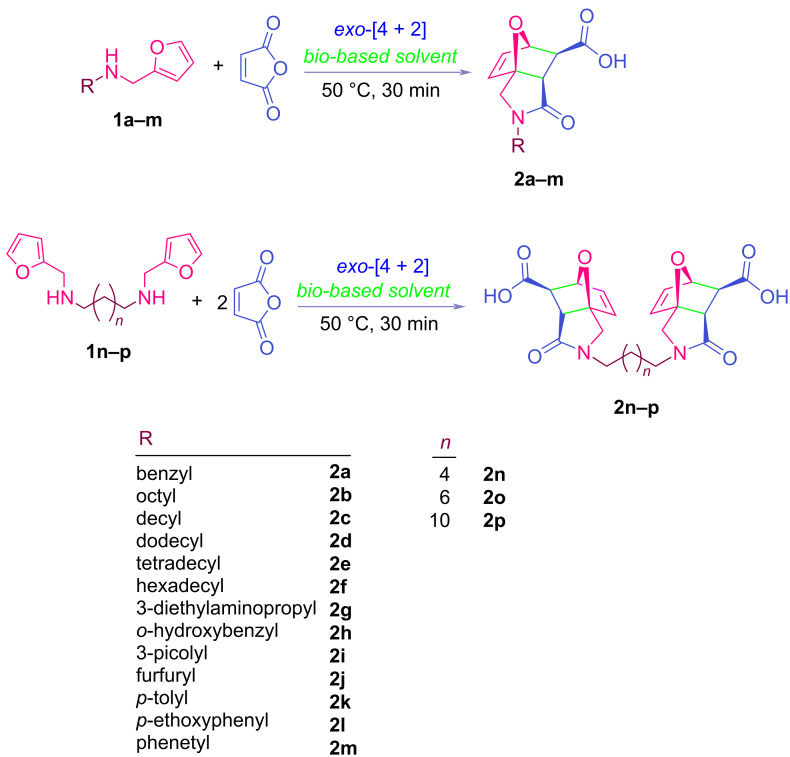
Synthesis of epoxyisoindole-7-carboxylic acids **2a–m** and **2n–p**.

According to X-ray and Raman spectroscopy techniques, triglyceride molecules in liquid form are arranged in a dynamic chain-like conformation [114–116]. As the temperature of the reaction medium increases, the size of these lamellar units decreases, increasing the ability to dissolve Diels–Alder components and intermediates. It should be noted that the hydrophobicity of triglycerides is much higher than that of aromatic solvents such as benzene and toluene. For comparison with vegetable oils, the reaction was also carried out in a conventional organic solvent such as benzene, and the yield of the corresponding product is given in Table 1, entry 8. The epoxyisoindolinone product **2a** formed by tandem intramolecular addition was easily separated from the reaction medium by precipitation with a hexane–ether mixture and was found to be of sufficient purity for spectroscopic analysis. If desired, the product can be crystallized from an EtOAc–MeOH mixture for further purification. On the other hand, compound **2a** has also been previously investigated for its potential covalent prolyl oligopeptidase inhibitory activity in human cells [117]. The solvent mixture used to precipitate the product was removed from the filtrate using a rotary evaporator and it was found that the used bio-based solvents could be recovered in a near quantitative amount for reuse. The recovered SSO solvent was reused as the reaction medium seven times under optimum conditions and the results for the cycloadduct **2k** are shown in Figure 1. As shown in the figure, there is no significant decrease in product yield. The reaction conditions and isolation method used in the literature procedures for the previous preparation of compound **2a** are given in Table S2 (Supporting Information File 1). As can be seen from the table, most of these methods use toxic and volatile organic solvents, and the duration of the associated intramolecular *exo*-[4 + 2] reaction is quite long. In the scope of this study, the structures and synthesis scheme of various epoxyisoindole-7-carboxylic acids synthesized using the reaction conditions given in Table 1, entry 4 are given in Table 2 and Scheme 1, respectively.

**Table 2:** Synthesized tricyclic epoxyisoindole-7-carboxylic acids^a^.

Compound	Structure	Yield (%) SSO	Yield (%) OO	Yield (%) OA	Yield (%) LM

**2a**	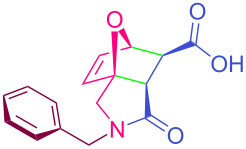	96	96	99	99
**2b**	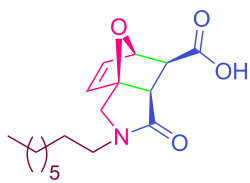	93	92	98	97
**2c**	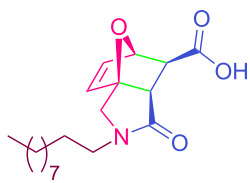	94	94	99	98
**2d**	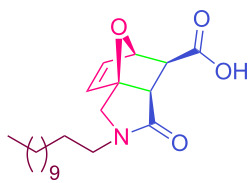	93	94	98	98
**2e**	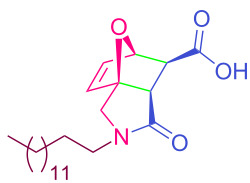	95	92	98	92
**2f**	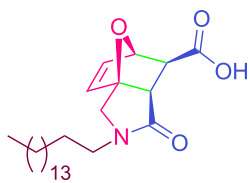	96	93	97	96
**2g**	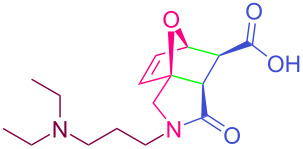	95	94	97	95
**2h**	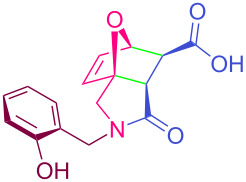	95	92	96	94
**2i**	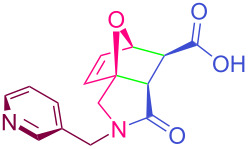	96	93	97	95
**2j**	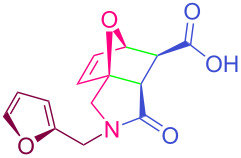	97	92	98	96
**2k**	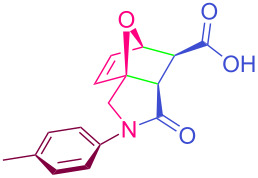	99	97	99	99
**2l**	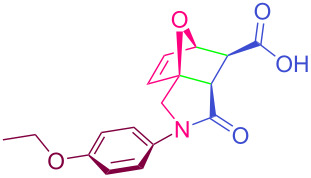	93	96	97	97
**2m**	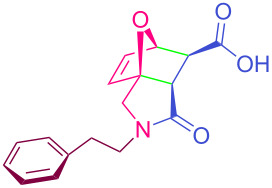	94	95	96	97
**2n**	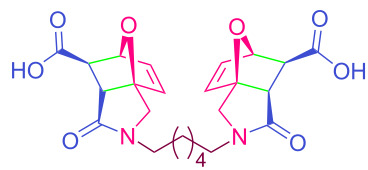	98	96	97	97
**2o**	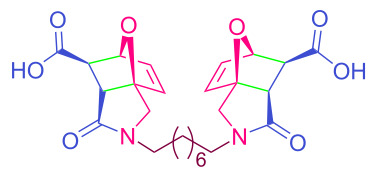	97	95	96	97
**2p**	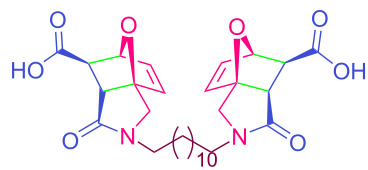	98	97	99	98

^a^Isolated yields.

**Scheme 2:**
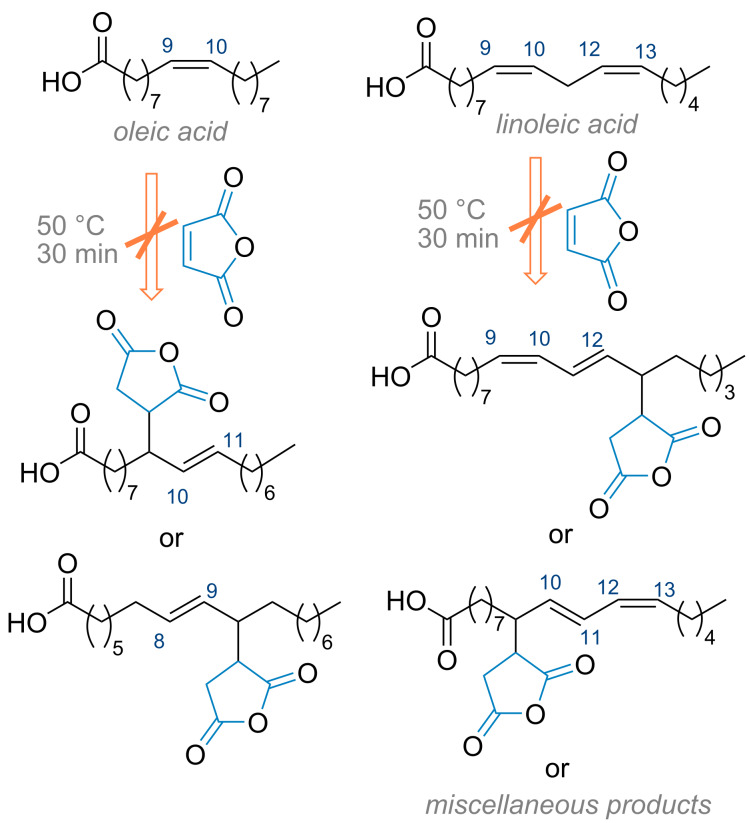
Possible side reactions of unsaturated fatty acids with maleic anhydride.

**Figure 2:**
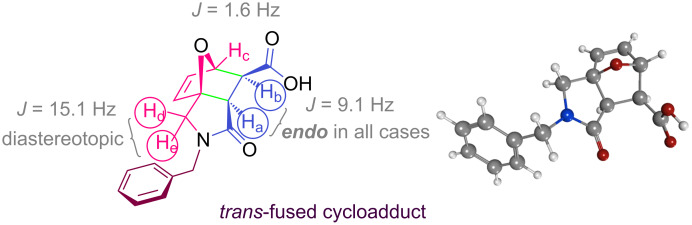
Coupling constants of selected protons in compound **2a** and its optimized geometric structure.

In general, IMDAF reactions proceed with high stereoselectivity to form the major *exo* addition product, in contrast to conventional Diels–Alder reactions [95,118–124]. On the other hand, the initially *exo* carboxylic acid substituent in the oxanorbornene ring is converted to its *endo* isomer by epimerization upon heating the cycloadduct in a mixture of pyridine–glacial acetic acid (1:1). In the process developed in this study, isolated double bonds in the fatty acid chains in sunflower oil do not undergo a side ene reaction with maleic anhydride, which requires high temperatures and/or metal catalysts, since the IMDAF reaction can be performed at 50 ºC or room temperature (Scheme 2) [125–126]. The olefin moiety in *N*-furfuryl-*N*-benzylmaleamic acid, formed by acylation of *N*-benzyl-*N*-furfurylamine with maleic anhydride, adopts a favorable conformational structure thanks to two activating electron-withdrawing groups and participates in the formation of the corresponding IMDAF product in oil medium rapidly and in good yields. The ring stereochemistry (*exo-* or *endo-*geometry) of the product, epoxyisoindole-7-carboxylic acid, is easily determined by ^1^H NMR by evaluating the coupling constants of the protons H_a_, H_b_ and H_c_ (Figure 2) [119,127–128].

**Scheme 3:**
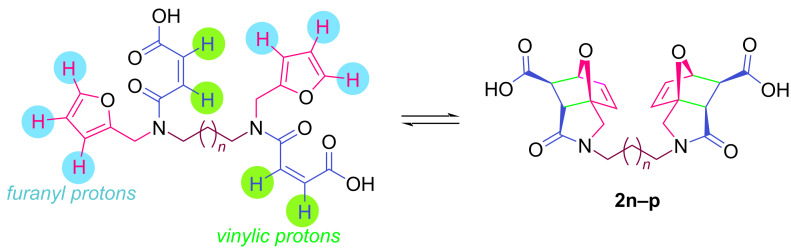
Equilibrium of **2n–p** with maleamic acid precursors.

The values of the coupling constants shown in Figure 2 are in good agreement with those reported for this compound in the literature. In all synthesized compounds, H_a_ and H_b_ protons are only in *endo* conformation and therefore the amide and carboxylic acid functional groups are arranged in *exo* conformation. The fact that the flexible **2n** and **2o** compounds in the bis structure are in equilibrium with maleamic acid precursors (Scheme 3) complicates the interpretation of their ^1^H and ^13^C NMR spectra. The existence of these precursors is clearly demonstrated by both their ^1^H and ^13^C NMR spectra (Supporting Information File 1). It should be noted that in the case of compound **2p**, unlike the other two compounds, the equilibrium is largely in favor of the product of interest and the NMR spectra confirm this.

From the mechanistic point of view of the IMDAF reaction, the presence of both *s-cis* and *s-trans* conformations of maleamic acid intermediates in the reaction medium has been previously demonstrated by ^1^H NMR studies [129]. The elongation of the alkyl chain attached to the N atom facilitates the formation of the epoxyisoindole product by favoring the *s-cis* conformation due to steric factors (*Thorpe–Ingold effect*) [130–131].

**Figure 3:**
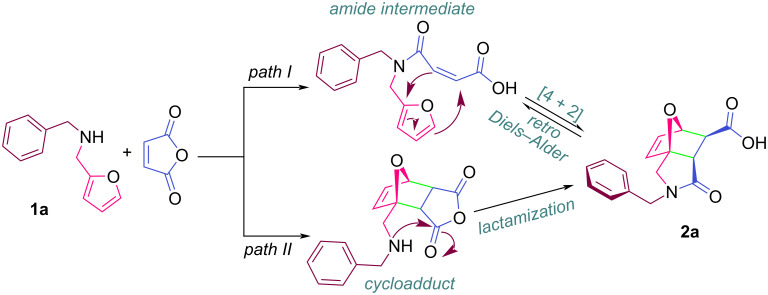
Possible mechanism for the IMDAF reaction.

Briefly, in this type of reaction, the acylation is followed by an intramolecular Diels–Alder reaction and the amide intermediate (a sample compound has been previously isolated and fully characterized as a result of detailed studies) is in equilibrium with the final product at room temperature in polar solvents such as DMSO (Figure 3, *path I*) [132]. Similarly, Tomberg et al., note that in such reactions, amide is formed rapidly by opening the maleic anhydride ring first, followed by a slower cyclization [133]. On the other hand, according to the results of some DFT calculations, Naguib et al., state that [4 + 2] cycloaddition between maleic anhydride and secondary amine occurs first, followed by the corresponding amidation (Figure 3, *path II*) [110].

**Figure 4:**
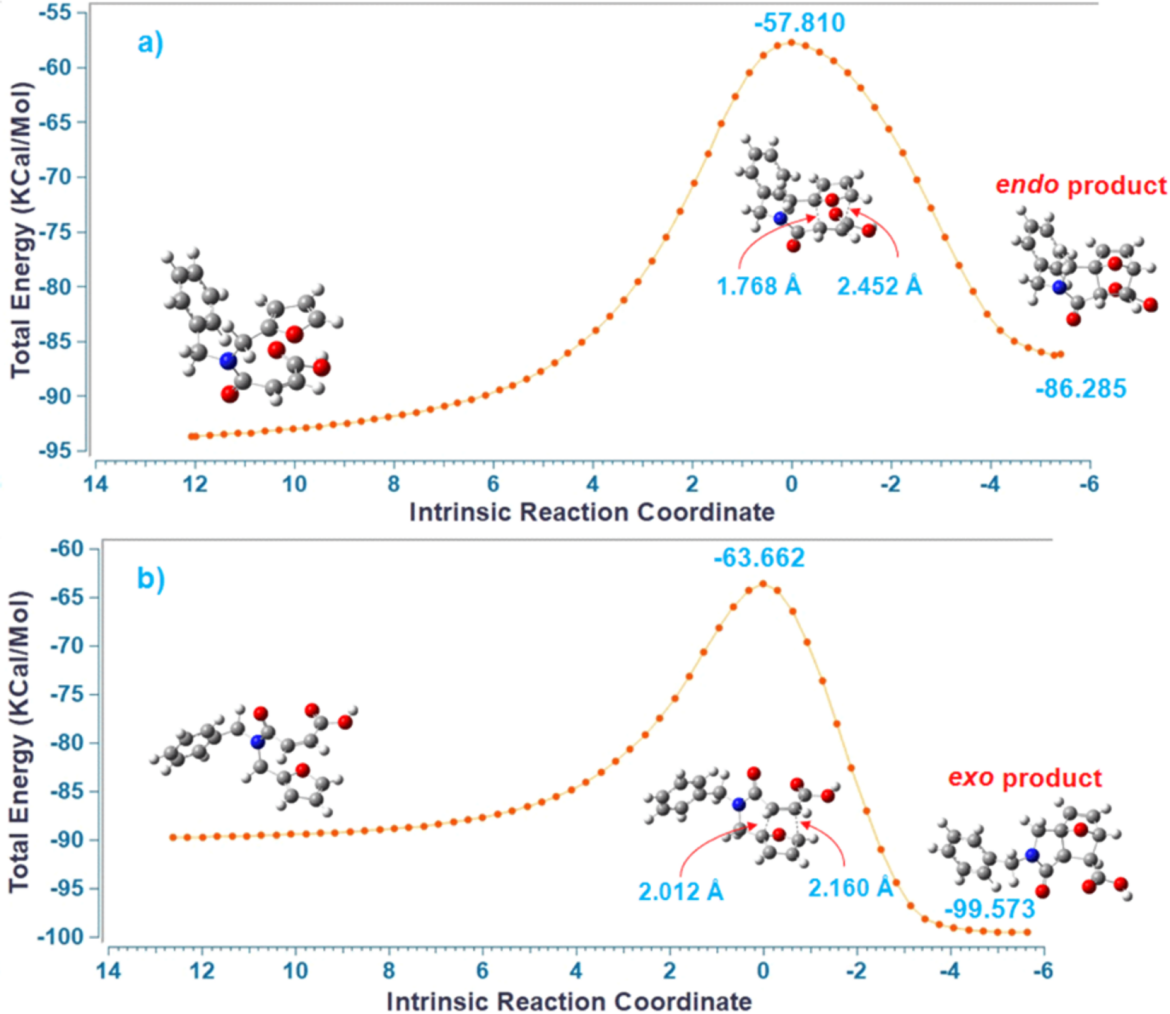
IRC calculations of a) *endo-*, b) *exo-*transition structures and products for compound **2a** (semi-empirical method, PM6) [136].

Although IMDAF reactions involving furan systems bearing amide functionality are generally reversible [134], the formation of the addition products in high yields in this study indicates that the *retro-*IMDAF reaction does not occur under the reaction conditions developed. As seen in Figure 4b, the different sigma bond lengths formed in the *exo* transition state (calculations which were performed in Gaussian 16 Rev B.01 (Gaussian, Inc., Wallingford, CT, USA)) [135] indicate that the IMDAF reaction proceeds through an asynchronous process (the same is true for the *endo* transition state). Accordingly, the length of the σ bond formed near the amide functional group was calculated to be 2.012 Å, while the length of the bond formed on the far side was calculated to be 2.160 Å. These bond lengths support *path I*, which is a more valid pathway in the reaction mechanism. As can be seen in Figure 4, the energies of both the *exo* transition state and the *exo* product are lower than those of the *endo*, which also supports the experimental results.

## Conclusion

Vegetable oils, which are readily available from a large number and variety of natural sources around the world, fatty acids and wax esters, can be excellent alternative reaction media to existing conventional and green solvents for IMDAF reactions. This study reveals the first utilization of these materials as biocompatible solvents for IMDAF reactions. The corresponding tricyclic epoxyisoindole-7-carboxylic acids were obtained in less time and in higher yields than in reactions carried out in conventional toxic and volatile organic solvents. A challenge in the use of vegetable oils as solvents in organic transformations is the purification of the synthesized product, since these oils are non-volatile and non-polar. However, in this study, the choice of these materials as green solvents allows convenient isolation of the cycloadducts by precipitation followed by filtration with hexane or diethyl ether. Thus, IMDAF reactions, which are a useful strategy for the preparation of many natural compounds and various organic intermediates, can be safely carried out under more environmentally friendly conditions.

## Experimental

All reagents and solvents were purchased from Merck (Merck, Darmstadt, Germany), Sigma-Aldrich (St. Louis, MO), or Acros Organics (Thermo Fisher Scientific, Geel, Belgium) and used without further purification. Thin-layer chromatography was performed using silica gel (60 F_254_, Merck, Darmstadt, Germany) plates. Melting points were recorded by Büchi melting point B-540 apparatus (Büchi Labortechnik AG in Flawil, Switzerland). The IR spectra were measured by Spectrum Two FT-IR spectrometer (PerkinElmer, Massachusetts, USA). The NMR spectra were measured using Bruker Ultrashield Plus Biospin 400 MHz NMR spectrometer and A600a Agilent DD2 600 MHz NMR spectrometer (Santa Clara, California, USA) and chloroform-*d* (CDCl_3_) or hexadeuterodimethyl sulfoxide (DMSO-*d*_6_) as a solvent. Chemical shifts (δ) are reported in ppm and *J* values in Hertz. The elemental analyses were performed using an LECO CHNS-932 elemental analyzer (Saint Joseph, MI, USA).

### Representative procedure for the IMDAF reaction to afford **2a**

In a 50 mL round bottom flask, *N*-benzyl-1-(furan-2-yl)methanamine (**1a**, 0.30 g, 1.60 mmol) was dissolved in 2 mL of SSO and heated in an oil bath to 50 °C. Then, into the observed solution, maleic anhydride (0.16 g, 1.63 mmol) was added and the resulted mixture was heated at 50 °C for 30 min. Thereafter, the reaction mixture was cooled to room temperature and the desired product, **2a** was precipitated after trituration with hexane. The obtained solid product was isolated by filtration under vacuum, washed with ether–hexane solvent mixture and dried at room temperature. If desired for further purification the solid product can be crystallized from EtOAc/MeOH solvent mixture. Beige crystalline solid, mp 171–172 °C (EtOAc/MeOH); ^1^H NMR (600 MHz, CDCl_3_) δ 7.33 (t, *J* = 7.6 Hz, 2H, Ar), 7.28 (d, *J* = 7.4 Hz, 1H, Ar), 7.24 (t, *J* = 7.4 Hz, 2H, Ar), 6.42–6.39 (m, 2H, H_f_, H_g_), 5.25 (d, *J* = 1.6 Hz, 1H, H_c_), 4.63 (d, *J* = 15.1 Hz, 1H, H_h_), 4.42 (d, *J* = 15.1 Hz, 1H, H_i_), 3.83 (d, *J* = 12 Hz, 1H, H_d_), 3.65 (d, *J* = 12 Hz, 1H, H_e_), 2.96 (d, *J* = 9.1 Hz, 1H, H_a_), 2.84 (d, *J* = 9.1 Hz, 1H, H_b_); ^13^C NMR (150 MHz, CDCl_3_) δ 173.10, 172.51, 137.22, 135.20, 134.83, 128.92, 127.98, 127.87, 88.79, 82.36, 50.72, 48.53, 47.03, 45.86; Anal calcd for C_16_H_15_NO_4_ (285.30): C, 67.36; H, 5.30; N, 4.91; found: C, 67.30; H, 5.26; N, 4.86.

## Supporting Information

File 1 Tables S1 and S2, Cartesian coordinates of the optimized structures and copies of NMR spectra.

## Data Availability

All data that supports the findings of this study is available in the published article and/or the supporting information to this article.
