# Supplementary material for: Sustainable tandem acylation/Diels–Alder reaction toward versatile tricyclic epoxyisoindole-7-carboxylic acids in renewable green solvents
Source: Beilstein J Org Chem. 2024 Jun 6;20:1308–19. doi: 10.3762/bjoc.20.114 (PMC11181201; doi:10.3762/bjoc.20.114)
Supplement: File 1 — Tables S1 and S2, Cartesian coordinates of the optimized structures and copies of NMR spectra. [file Beilstein_J_Org_Chem-20-1308-s001.pdf]

BEILSTEIN JOURNAL OF ORGANIC CHEMISTRY

Supporting Information

for

Sustainable tandem acylation/Diels–Alder reaction toward versatile tricyclic epoxyisoindole-7-carboxylic acids in renewable green solvents

Ayhan Yıldırım

Beilstein J. Org. Chem. **2024,** *20,* 1308–1319. doi:10.3762/bjoc.20.114

Tables S1 and S2, Cartesian coordinates of the optimized structures and copies of NMR spectra

License and Terms: This is a supporting information file under the terms of the Creative Commons Attribution License (https://creativecommons.org/licenses/by/4.0). Please note that the reuse, redistribution and reproduction in particular requires that the author(s) and source are credited and that individual graphics may be subject to special legal provisions.
The license is subject to the *Beilstein Journal of Organic Chemistry* terms and conditions: (https://www.beilstein-journals.org/bjoc/terms)

Table of contents

Table S1……………………………………………………………………….S2

Table S2……………………………………………………………………….S3–S4

References for Tables S1 and S2……………………………………………S5–S6

Characterization data…………………………………………………………S7–S19

^{1}H NMR Spectra…………………………………………………………….S20–S35

^{13}C NMR Spectra……………………………………………………………..S36–S51

Cartesian coordinates of the optimized structures……………………………..S52–S63

Table S1: Composition and physical parameters of the used vegetable oils, oleic acid and wax ester.

Parameters (%)	SSO	OO
Saturated fatty acids	14	16
Monounsaturated fatty acids	29	65
Polyunsaturated fatty acids	57	10
Carbohydrate	0	0
Protein	0	0
Salt	0	0
OA		
Technical grade: 90%		
Vapour pressure 1 mmHg (176 °C)		
Refractive index, n20/D: 1.459		
Boiling point: 194-195 °C/1.2 mmHg		
Melting point: 13-14 °C		
Density: 0.887 g/mL at 25 °C		
LM		
Name: Lauryl myristate		
Analytical grade		
Melting point: 38–39 °C		
Synthesized according to ref. [1]		

Table S2: Literature methods given in literature for the cyclization of *N*-benzyl-1-(furan-2-yl)methanamine **1a** with maleic anhydride to afford **2a**.

Entry	Solvent	Temperature (°C)	Time	Product isolation	Yield (%)	Ref.
1	benzene	–	–	recrystallization (AcOH)	99.5	[2]
2	benzene	rt	48-72 h	filtration and washing with benzene	90	[3]
3	benzene	rt	10 h	recrystallization (hexane-EtOAc)	95	[4]
4	toluene	MW, 60	10 min	filtration and washing with ether	76	[5]
5	benzene	rt	24 h	filtration and washing with benzene-ether	90	[6]
6	toluene	rx	5 h	filtration and washing with ether	86	[7]
7	THF or toluene	rx-rt	24 h	filtration and washing with ether	-	[8]
8	diethyl ether	rt	24 h	filtration and washing with ether	-	[9]
9	toluene	rt	16 h	recrystallization (acetone)	55	[10]
10	chloroform	rt	48 h	filtration and	75	[11]

				washing with MeOH or acetone		
11	chloroform	rt-rx	7 h	filtration and washing with acetone	80	[12]
12	SSO	50	30 min	filtration and washing with hexane-ether	96	this work
13	OO	50	30 min	filtration and washing with hexane-ether	96	this work
14	OA	50	30 min	filtration and washing with hexane-ether	99	this work
15	LM	50	30 min	filtration and washing with hexane-ether	99	this work

References for Tables S1 and S2

1. Yıldırım, A.; Mudaber, S.; Öztürk, S. *Eur. J. Lipid Sci. Technol.* 2019, *121,* 1800303. doi: 10.1002/ejlt.201800303
2. Takano S.; Oshima Y.; Ito F.; Ogasawara K. *Yakugaku Zasshi* 1980, *100,* 1194–1202. doi: 10.1248/yakushi1947.100.12_1194
3. Varlamov, A. V.; Boltukhina, E. V.; Zubkov, F. I.; Sidorenko, N. V.; Chernyshev, A. I.; Grudinin, D. G. *Chem. Heterocycl. Compd.* **2004**, *40,* 22–28. doi: 10.1023/B:COHC.0000023763.75894.63
4. Sarang, P. S.; Yadava, A. A.; Patila, P. S.; Krishna, U. M.; Trivedi, G. K.; Salunkhe, M. M. *Synthesis* **2007**, 7, 1091–1095. doi: 10.1055/s-2007-965950
5. Gordon, C. P.; Byrne, N.; McCluskey, A. *Green Chem.* **2010**, *12,* 1000–1006. doi: 10.1039/B924835H
6. Zubkov, F. I.; Airiyan, I. K.; Ershova, J. D.; Galeev, T. R.; Zaytsev, V. P.; Nikitina, E. V.; Varlamov, A. V. *RSC Adv.* **2012,** *2,* 4103–4109. doi: 10.1039/C2RA20295F
7. De Cesco, S.; Deslandes, S.; Therrien, E.; Levan, D.; Cueto, M.; Schmidt, R.; Cantin, L. D.; Mittermaier, A.; Jeanneret, L. J.; Moitessier, N. *J. Med. Chem.* **2012**, *55,* 6306–6315. doi: 10.1021/jm3002839
8. Papeo, G.; Posteri, H.; Borghi, D.; Busel, A. A.; Caprera, F.; Casale, E.; Ciomei, M.; Cirla, A.; Corti, E.; D'Anello, M.; Fasolini, M.; Forte, B.; Galvani, A.; Isacchi, A.; Khvat, A.; Krasavin, M. Y.; Lupi, R.; Orsini, P.; Perego, R.; Pesenti, E.; Pezzetta, D.; Rainoldi, S.; Sirtori, F. R.; Scolaro, .; Sola, F.; Zuccotto, F.; Felder, E. R.; Donati, D.; Montagnoli, A. *J. Med. Chem.* **2015**, *58,* 6875–6898. doi: 10.1021/acs.jmedchem.5b00680

9. Hizartzidis, L.; Gilbert, J.; Gordon, C. P.; Sakoff, J. A.; McCluskey, A. *ChemMedChem* **2019**, *14,* 1152–1161. doi: 10.1002/cmdc.201900180

10. Blanpain, A.; Clark, J. H.; Farmer, T. J.; Guo, Y.; Ingram, I. D. V.; Kendrick, J. E.; Lawrenson, S. B.; North, M.; Rodgers, G.; Whitwood, A. C. *ChemSusChem* **2019,** *12,* 2393–2401. doi: 10.1002/cssc.201900748

11. Naguib, M.; Rashed, A.; Keddie, D. J. *J. Mater. Sci.* **2021**, *56,* 8900–8909. doi: 10.1007/s10853-021-05853-x

12. Naguib, M.; Yassin, M. A. *ACS Appl. Polym. Mater.* **2022**, *4,* 2181–2188. doi: 10.1021/acsapm.2c00158

2-Benzyl-1-oxo-1,2,3,6,7,7a-hexahydro-3a,6-epoxyisoindole-7-carboxylic acid ***(2a)***

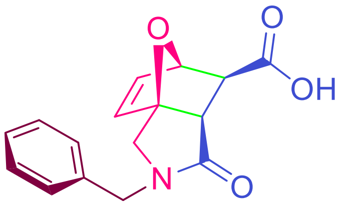

Beige crystalline solid, mp 171–172 °C (EtOAc/MeOH), (The analytical data of compound **2a** agree with the literature [6], lit. 170.3–171.8 °C); ^{1}H NMR (600 MHz, $CDCl_3$) δ 7.33 (*t*, *J* = 7.6 Hz, 2H, Ar), 7.28 (*d*, *J* = 7.4 Hz, 1H, Ar), 7.24 (*t*, *J* = 7.4 Hz, 2H, Ar), 6.42-6.39 (*m*, 2H, H_f, H_g), 5.25 (*d*, *J* = 1.6 Hz, 1H, H_c), 4.63 (*d*, *J* = 15.1 Hz, 1H, H_h), 4.42 (*d*, *J* = 15.1 Hz, 1H, H_i), 3.83 (*d*, *J* = 12 Hz, 1H, H_d), 3.65 (*d*, *J* = 12 Hz, 1H, H_e), 2.96 (*d*, *J* = 9.1 Hz, 1H, H_a), 2.84 (*d*, *J* = 9.1 Hz, 1H, H_b); ^{13}C NMR (150 MHz, $CDCl_3$) δ 173.10, 172.51, 137.22, 135.20, 134.83, 128.92, 127.98, 127.87, 88.79, 82.36, 50.72, 48.53, 47.03, 45.86; Anal calc for $C_{16}H_{15}NO_4$ (285.30): C 67.36, H 5.30, N 4.91. Found: C 67.30, H 5.26, N 4.86.

[6] Zubkov, F. I.; Airiyan, I. K.; Ershova, J. D.; Galeev, T. R.; Zaytsev, V. P.; Nikitina, E. V.; Varlamov, A. V. *RSC Adv.* **2012,** *2,* 4103–4109. doi: 10.1039/C2RA20295F

2-Octyl-1-oxo-1,2,3,6,7,7a-hexahydro-3a,6-epoxyisoindole-7-carboxylic acid ***(2b)***

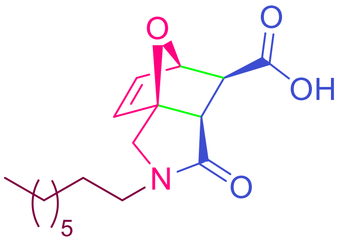

Beige crystalline solid, mp 119–120 °C (EtOAc/MeOH); ^{1}H NMR (600 MHz, $CDCl_3$) δ 6.47 (*s*, 2H, H_f, H_g), 5.28 (*s*, 1H, H_c), 3.98 (*d*, *J* = 11.9 Hz, 1H, H_d), 3.73 (*d*, *J* = 11.9 Hz, 1H, H_e), 3.44-3.40 (*m*, 1H, H_h), 3.24-3.19 (*m*, 1H, H_i), 2.89 (*d*, *J* = 9.1 Hz, 1H, H_a), 2.82 (*d*, *J* = 9.1 Hz, 1H, H_b), 1.52 (*quin*, *J* = 7.6 Hz, 2H, -$NCH_2C\underline{H}_2CH_2$-), 1.27-1.23 (*m*, 10H, $5CH_2$), 0.85 (*t*, *J* = 7.2 Hz, 3H, -CH_3); ^{13}C NMR (150 MHz, $CDCl_3$) δ 172.88, 172.52, 137.30, 134.77, 88.94, 82.49, 50.51, 49.00, 46.05, 43.25, 31.73, 29.14, 29.10, 27.02, 26.62, 22.58, 14.04; Anal calc for $C_{17}H_{25}NO_4$ (307.39): C 66.43, H 8.20, N 4.56. Found: C 66.38, H 8.28, N 4.47.

2-Decyl-1-oxo-1,2,3,6,7,7a-hexahydro-3a,6-epoxyisoindole-7-carboxylic acid ***(2c)***

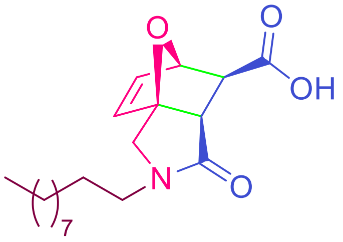

Beige crystalline solid, mp 103–104 °C (EtOAc/MeOH); ^{1}H NMR (600 MHz, $CDCl_3$) δ 6.47 (*s*, 2H, H_f, H_g), 5.29 (*s*, 1H, H_c), 3.98 (*d*, *J* = 11.9 Hz, 1H, H_d), 3.73 (*d*, *J* = 11.9 Hz, 1H, H_e), 3.44-3.40 (*m*, 1H, H_h), 3.24-3.19 (*m*, 1H, H_i), 2.89 (*d*, *J* = 9.1 Hz, 1H, H_a), 2.83 (*d*, *J* = 9.1 Hz, 1H, H_b), 1.52 (*quin*, *J* = 7.2 Hz, 2H, -$NCH_2C\underline{H}_2CH_2$-), 1.27-1.23 (*m*, 14H, $7CH_2$), 0.86 (*t*, *J* = 7.4 Hz, 3H, -CH_3); ^{13}C NMR (150 MHz, $CDCl_3$) δ 172.89,

172.52, 137.30, 134.77, 88.94, 82.49, 50.51, 49.00, 46.05, 43.25, 31.84, 29.49, 29.45, 29.25, 29.19, 27.03, 26.63, 22.63, 14.07; Anal calc for $C_{19}H_{29}NO_4$ (335.44): C 68.03, H 8.71, N 4.18. Found: C 68.08, H 8.64, N 4.23.

2-Dodecyl-1-oxo-1,2,3,6,7,7a-hexahydro-3a,6-epoxyisoindole-7-carboxylic acid ***(2d)***

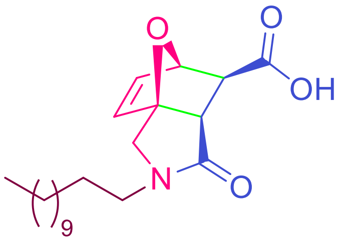

Beige crystalline solid, mp 103.5–104 °C (EtOAc/MeOH); ^{1}H NMR (600 MHz, $CDCl_3$) δ 6.46 (*s*, 2H, H_f, H_g), 5.27 (*s*, 1H, H_c), 3.98 (*d*, *J* = 11.9 Hz, 1H, H_d), 3.74 (*d*, *J* = 11.9 Hz, 1H, H_e), 3.46-3.39 (*m*, 1H, H_h), 3.23-3.17 (*m*, 1H, H_i), 2.89 (*d*, *J* = 9.1 Hz, 1H, H_a), 2.83 (*d*, *J* = 9.1 Hz, 1H, H_b), 1.52 (*quin*, *J* = 7.2 Hz, 2H, -$NCH_2C\underline{H}_2CH_2$-), 1.28-1.22 (*m*, 18H, 9CH_2), 0.85 (*t*, *J* = 7.4 Hz, 3H, -CH_3); ^{13}C NMR (150 MHz, $CDCl_3$) δ 173.12, 172.47, 137.25, 134.81, 88.90, 82.41, 50.64, 48.99, 45.91, 43.26, 31.87, 29.60, 29.59, 29.55, 29.45, 29.30, 29.21, 27.04, 26.65, 22.64, 14.07; Anal calc for $C_{21}H_{33}NO_4$ (363.50): C 69.39, H 9.15, N 3.85. Found: C 69.45, H 9.09, N 3.91.

2-Tetradecyl-1-oxo-1,2,3,6,7,7a-hexahydro-3a,6-epoxyisoindole-7-carboxylic acid ***(2e)***

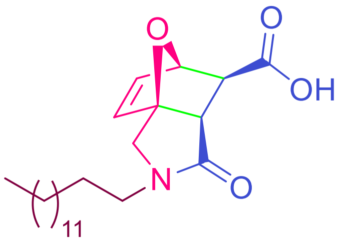

Beige crystalline solid, mp 112–113 °C (EtOAc/MeOH); ^{1}H NMR (600 MHz, $CDCl_3$) δ 6.47 (*s*, 2H, H_f, H_g), 5.31 (*s*, 1H, H_c), 3.98 (*d*, *J* = 11.9 Hz, 1H, H_d), 3.74 (*d*, *J* = 11.9 Hz, 1H, H_e), 3.45-3.40 (*m*, 1H, H_h), 3.25-3.20 (*m*, 1H, H_i), 2.89 (*d*, *J* = 9.1 Hz, 1H, H_a), 2.84 (*d*, *J* = 9.1 Hz, 1H, H_b), 1.53 (*quin*, *J* = 7.2 Hz, 2H, -$NCH_2C\underline{H}_2CH_2$-), 1.27-1.23 (*m*, 22H, 11CH_2), 0.86 (*t*, *J* = 7.2 Hz, 3H, -CH_3); ^{13}C NMR (150 MHz, $CDCl_3$) δ 172.73, 172.57, 137.37, 134.70, 88.99, 82.57, 50.39, 49.02, 46.21, 43.27, 31.89, 29.66, 29.64, 29.61, 29.56, 29.46, 29.32, 29.20, 27.03, 26.64, 22.65, 14.08; Anal calc for $C_{23}H_{37}NO_4$ (391.55): C 70.55, H 9.53, N 3.58. Found: C 70.50, H 9.48, N 3.66.

2-Hexadecyl-1-oxo-1,2,3,6,7,7a-hexahydro-3a,6-epoxyisoindole-7-carboxylic acid ***(2f)***

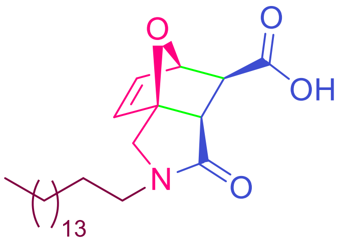

Beige crystalline solid, mp 114–115 °C (EtOAc/MeOH); ^{1}H NMR (600 MHz, $CDCl_3$) δ 6.47 (*s*, 2H, H_f, H_g), 5.31 (*s*, 1H, H_c), 3.98 (*d*, *J* = 11.9 Hz, 1H, H_d), 3.74 (*d*, *J* = 11.9 Hz, 1H, H_e), 3.45-3.40 (*m*, 1H, H_h), 3.25-3.20 (*m*, 1H, H_i), 2.89 (*d*, *J* = 9.1 Hz, 1H, H_a),

2.84 (*d*, *J* = 9.1 Hz, 1H, H_b), 1.53 (*quin*, *J* = 7.2 Hz, 2H, $-NCH_2C\underline{H}_2CH_2-$), 1.27-1.23 (*m*, 26H, $13CH_2$), 0.86 (*t*, *J* = 7.2 Hz, 3H, $-CH_3$); ^{13}C NMR (150 MHz, $CDCl_3$) δ 172.68, 172.58, 137.39, 134.67, 89.00, 82.60, 50.35, 49.02, 46.26, 43.28, 31.89, 29.66, 29.63, 29.61, 29.56, 29.46, 29.33, 29.20, 27.03, 26.64, 22.65, 14.08; Anal calc for $C_{25}H_{41}NO_4$ (419.61): C 71.56, H 9.85, N 3.34. Found: C 71.50, H 9.80, N 3.29.

2-(3-(Diethylamino)propyl)-1-oxo-1,2,3,6,7,7a-hexahydro-3a,6-epoxyisoindole-7-carboxylic acid ***(2g)***

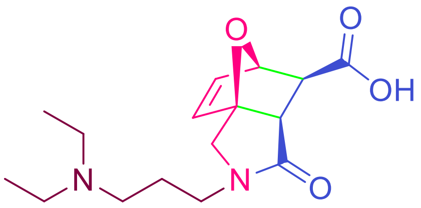

Orange oily liquid; ^{1}H NMR (600 MHz, DMSO-d_6) δ 6.53 (*d*, *J* = 5.9 Hz, 1H, H_f), 6.39 (*d*, *J* = 5.9 Hz, 1H, H_g), 4.91 (*s*, 1H, H_c), 3.96 (*d*, *J* = 11.6 Hz, 1H, H_d), 3.54 (*d*, *J* = 11.6 Hz, 1H, H_e), 3.22-3.19 (*m*, 1H, H_h), 3.16-3.12 (*m*, 1H, H_i), 2.67 (*d*, *J* = 9.2 Hz, 1H, H_a), 2.46 (*q*, 4H, *J* = 13.2 Hz,$-NCH_2CH_2CH_2N(C\underline{H}_2CH_3)_2$), 2.40 (*d*, *J* = 9.2 Hz, 1H, H_b), 1.59 (*t*, *J* = 7.4 Hz, 2H, $-NCH_2CH_2C\underline{H}_2N(CH_2CH_3)_2$), 1.02 (*quin*, 2H, *J* = 7.2 Hz, $-NCH_2C\underline{H}_2CH_2N(CH_2CH_3)_2$), 0.95 (*t*, *J* = 7.3 Hz, 6H, $-NCH_2CH_2CH_2N(CH_2C\underline{H}_3)_2$); ^{13}C NMR (150 MHz, DMSO-d_6) δ 172.68, 172.58, 137.39, 134.67, 89.00, 82.60, 56.47, 50.62, 49.58, 48.14, 46.42, 45.29, 24.06, 18.96, 11.20; Anal calc for $C_{16}H_{24}N_2O_4$ (308.38): C 62.32, H 7.84, N 9.08. Found: C 62.28, H 7.90, N 9.13.

2-(2-Hydroxybenzyl)-1-oxo-1,2,3,6,7,7a-hexahydro-3a,6-epoxyisoindole-7-carboxylic acid ***(2h)***

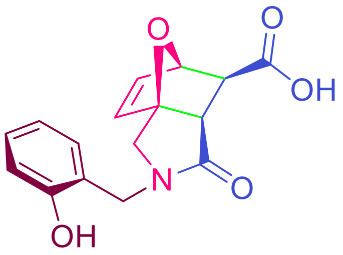

White crystalline solid, mp 103–104 °C (EtOAc/MeOH); ^{1}H NMR (600 MHz, $CDCl_3$) δ 7.19 (*t*, *J* = 9.7 Hz, 1H, Ar), 7.11 (*d*, *J* = 9.7 Hz, 1H, Ar), 6.92 (*d*, *J* = 7.5 Hz, 1H, Ar), 6.82 (*t*, *J* = 7.5 Hz, 1H, Ar), 6.44 (*d*, *J* = 5.9 Hz, 1H, H_g), 6.41 (*dd*, J_1 = 5.9 Hz, J_2 = 1.7 Hz, 1H, H_f), 5.20 (*d*, *J* = 1.7 Hz, 1H, H_c), 4.75 (*d*, *J* = 14.9 Hz, 1H, H_h), 4.12 (*d*, *J* = 14.9 Hz, 1H, H_i), 3.97 (*d*, *J* = 12 Hz, 1H, H_d), 3.79 (*d*, *J* = 12 Hz, 1H, H_e), 2.86 (*d*, *J* = 9.1 Hz, 1H, H_a), 2.78 (*d*, *J* = 9.1 Hz, 1H, H_b); ^{13}C NMR (150 MHz, $CDCl_3$) δ 174.36, 173.41, 155.53, 136.89, 135.17, 130.81, 130.21, 121.30, 119.95, 117.49, 88.82, 82.13, 51.32, 49.32, 45.07, 44.07; Anal calc for $C_{16}H_{15}NO_5$ (301.30): C 63.78, H 5.02, N 4.65. Found: C 63.84, H 5.07, N 4.59.

1-Oxo-2-(pyridin-3-ylmethyl)-1,2,3,6,7,7a-hexahydro-3a,6-epoxyisoindole-7-carboxylic acid ***(2i)***

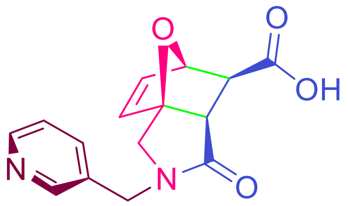

Yellowish solid, mp 183–184 °C (dec.) (EtOAc/MeOH); ^{1}H NMR (600 MHz, $CDCl_3$) δ 8.58-8.56 (*m*, 2H, Py), 7.64 (*d*, *J* = 5.7 Hz, 1H, Py), 7.32-7.30 (*m*, 1H, Py), 6.47 (*d*, *J* = 2.6 Hz, 2H, H_f, H_g), 5.29 (*d*, *J* = 1.4 Hz, 1H, H_c), 4.67 (*d*, *J* = 15.4 Hz, 1H, H_h), 4.44 (*d*, *J* = 15.4 Hz, 1H, H_i), 3.91 (*d*, *J* = 11.8 Hz, 1H, H_d), 3.66 (*d*, *J* = 11.8 Hz, 1H, H_e), 2.87 (*d*, *J* = 9.1 Hz, 1H, H_a), 2.83 (*d*, *J* = 9.1 Hz, 1H, H_b); ^{13}C NMR (150 MHz, $CDCl_3$) δ 172.93, 172.22, 148.58, 137.28, 136.28, 134.74, 131.62, 128.31, 124.06, 88.77, 82.32, 50.48, 48.52, 46.02, 44.31; Anal calc for $C_{15}H_{14}N_2O_4$ (286.29): C 62.93, H 4.93, N 9.79. Found: C 62.88, H 4.98, N 9.85.

*2-(Furan-2-ylmethyl)-1-oxo-1,2,3,6,7,7a-hexahydro-3a,6-epoxyisoindole-7-carboxylic acid **(2j)***

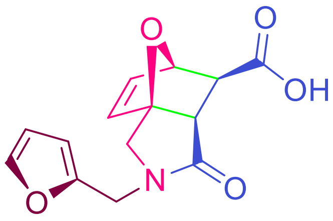

Beige crystalline solid, mp 148–150 °C (EtOAc/MeOH), (The analytical data of compound **2j** agree with the literature [13], lit. 150–151 °C); ^{1}H NMR (600 MHz, $CDCl_3$) δ 7.35 (*dd*, J_1 = 1.9 Hz, J_2 = 0.9 Hz, 1H, Furyl), 6.44 (*m*, 2H, H_f, H_g), 6.31 (*dd*, J_1 = 3.2 Hz, J_2 = 1.8 Hz, 1H, Furyl), 6.28 (*dd*, J_1 = 3.2 Hz, J_2 = 0.9 Hz, 1H, Furyl), 5.24 (*d*, *J* = 1.6 Hz, 1H, H_c), 4.75 (*d*, *J* = 15.5 Hz, 1H, H_h), 4.26 (*d*, *J* = 15.5 Hz, 1H, H_i), 3.92 (*d*, *J* = 12 Hz, 1H, H_d), 3.76 (*d*, *J* = 12 Hz, 1H, H_e), 2.90 (*d*, *J* = 9.1 Hz, 1H, H_a), 2.81 (*d*, *J* = 9.1 Hz, 1H, H_b); ^{13}C NMR (150 MHz, $CDCl_3$) δ 173.47, 172.04, 149.06, 142.67, 137.15, 134.95, 110.58, 108.84, 88.72, 82.23, 50.72, 48.92, 45.60, 39.86; Anal calc for $C_{14}H_{13}NO_5$ (275.26): C 61.09, H 4.76, N 5.09. Found: C 61.03, H 4.81, N 5.16.

[13] Zaytsev, V. P.; Mikhailova, N. M.; Airiyan, I. K.; Galkina, E. V.; Golubev, V. D.; Nikitina, E. V.; Zubkov, F. I; Varlamov, A. V. *Chem. Heterocycl. Compd.* **2012**, *48*, 505–513. doi: 10.1007/s10593-012-1023-1

1-Oxo-2-(p-tolyl)-1,2,3,6,7,7a-hexahydro-3a,6-epoxyisoindole-7-carboxylic acid ***(2k)***

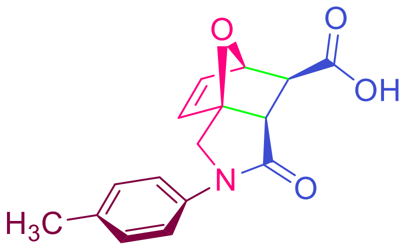

White crystalline solid, mp 188–190 °C (EtOAc/MeOH), (The analytical data of compound **2k** agree with the literature [14], lit. 183–184 °C); ^{1}H NMR (600 MHz, $CDCl_3$) δ 7.43 (*d*, *J* = 8.5 Hz, 2H, Ar), 7.18 (*d*, *J* = 8.5 Hz, 2H, Ar), 6.56 (*d*, *J* = 1 Hz, 2H, H_f, H_g), 5.41 (*s*, 1H, H_c), 4.47 (*d*, *J* = 11.8 Hz, 1H, H_d), 4.21 (*d*, *J* = 11.8 Hz, 1H, H_e), 3.05 (*d*, *J* = 9.1 Hz, 1H, H_a), 2.95 (*d*, *J* = 9.1 Hz, 1H, H_b), 2.33 (*s*, 3H, CH_3); ^{13}C NMR (150 MHz, $CDCl_3$) δ 171.97, 171.42, 144.52, 137.49, 135.70, 134.63, 129.58, 121.01, 88.30, 82.74, 51.14, 50.76, 46.88, 20.09; Anal calc for $C_{16}H_{15}NO_4$ (285.10): C 67.36, H 5.30, N 4.91. Found: C 67.41, H 5.25, N 4.85.

[14] Bilović, D. *Croat. Chem. Acta* **1966**, *38,* 293–298. doi: https://hrcak.srce.hr/208149

2-(4-Ethoxyphenyl)-1-oxo-1,2,3,6,7,7a-hexahydro-3a,6-epoxyisoindole-7-carboxylic acid ***(2l)***

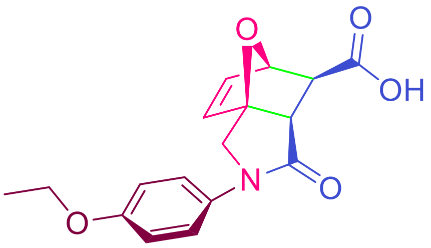

White crystalline solid, mp 171–172 °C (EtOAc/MeOH); ^{1}H NMR (600 MHz, DMSO-d_6) δ 7.51 (*d*, *J* = 9.1 Hz, 2H, Ar), 6.90 (*d*, *J* = 9.1 Hz, 2H, Ar), 6.61 (*d*, *J* = 5.6 Hz, 1H, H_g), 6.45 (*dd*, J_1 = 5.6 Hz, J_2 = 1.7 Hz, 1H, H_f), 5.01 (*d*, J_1 = 1.7 Hz, 1H, H_c), 4.48 (*d*, *J* = 12 Hz, 1H, H_d), 3.99 (*d*, *J* = 12 Hz, 1H, H_e), 4.00-3.96 (*m*, 2H, -OC$\underline{H}_2$CH$_3$), 3.00 (*d*, *J* = 9.1 Hz, 1H, H_a), 2.54 (*d*, *J* = 9.1 Hz, 1H, H_b), 1.28 (*t*, *J* = 7.1 Hz, 3H, -OCH$_2$C$\underline{H}_3$); ^{13}C NMR (150 MHz, DMSO-d_6) δ 173.39, 170.30, 155.45, 137.36, 135.87, 133.05, 121.54, 114.77, 87.85, 81.75, 63.58, 51.78, 49.88, 45.60, 15.08; Anal calc for $C_{17}H_{17}NO_5$ (315.33): C 64.75, H 5.43, N 4.44. Found: C 64.70, H 5.38, N 4.50.

1-Oxo-2-phenethyl-1,2,3,6,7,7a-hexahydro-3a,6-epoxyisoindole-7-carboxylic acid ***(2m)***

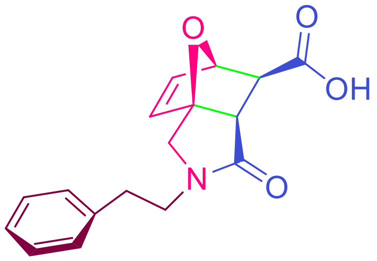

Beige crystalline solid, mp 116–117 °C (EtOAc/MeOH), (lit. 89–90 °C, ref.[4]; ^{1}H NMR (600 MHz, $CDCl_3$) δ 7.28 (*t*, *J* = 7.6 Hz, 2H, Ar), 7.22-7.19 (*d*, *J* = 7.4 Hz, 3H, Ar), 6.42-6.39 (*m*, 2H, H_f, H_g), 5.24 (*d*, *J* = 1.7 Hz, 1H, H_c), 3.77 (*d*, *J* = 13.9 Hz, 1H, H_h), 3.62 (*d*, *J* = 13.9 Hz, 1H, H_i), 3.74-3.69 (*m*, 1H, H_d), 3.45-3.40 (*m*, 1H, H_e), 2.91-2.81 (*m*, 2H, -$CH_2C\underline{H}_2$Ph), 2.96 (*d*, *J* = 9.1 Hz, 1H, H_a), 2.84 (*d*, *J* = 9.1 Hz, 1H, H_b); ^{13}C NMR (150 MHz, $CDCl_3$) δ 173.19, 172.47, 138.30, 137.11, 134.91, 128.71, 128.66, 126.63, 88.87, 82.29, 50.68, 49.74, 45.71, 44.86, 33.54; Anal calc for $C_{17}H_{17}NO_4$ (299.33): C 68.22, H 5.72, N 4.68. Found: C 68.17, H 5.76, N 4.74.

7-Carboxy-1-oxo-1,6,7,7a-tetrahydro-3a,6-epoxyisoindol-2(3H)-yl)hexyl)-1-oxo-1,2,3,6,7,7a-hexahydro-3a,6-epoxyisoindole-7-carboxylic acid ***(2n)***

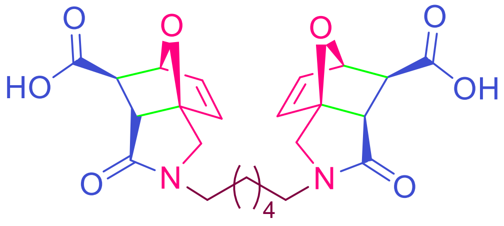

Orange solid, mp 68–69 °C; ^{1}H NMR (600 MHz, DMSO-d_6) δ 6.55-6.53 (*m*, 2H, H_g), 6.40-6.38 (*m*, 2H, H_f), 4.93 (*s*, 2H, H_c), 3.95 (*d*, *J* = 11.7 Hz, 2H, H_d), 3.83 (*d*, *J* = 11.9 Hz, 2H, H_e), 3.53-3.50 (*m*, 2H, H_h), 3.26-3.16 (*m*, 2H, H_i), 3.12-3.03 (*m*, 2H, H_a), 2.75-

2.70 (*m*, 2H, H$_b$), 1.45-1.38 (*m*, 4H, 2CH$_2$), 1.27-1.19 (*m*, 4H, 2CH$_2$); ^{13}C NMR (150 MHz, DMSO-d$_6$) δ 173.44, 170.70, 137.09, 136.10, 88.80, 81.52, 50.64, 47.96, 44.98, 26.96, 25.99, 25.84; Anal calc for $C_{24}H_{28}N_2O_8$ (472.49): C 61.01, H 5.97, N 5.93. Found: C 61.08, H 5.90, N 5.86.

7-Carboxy-1-oxo-1,6,7,7a-tetrahydro-3a,6-epoxyisoindol-2(3H)-yl)octyl)-1-oxo-1,2,3,6,7,7a-hexahydro-3a,6-epoxyisoindole-7-carboxylic acid ***(2o)***

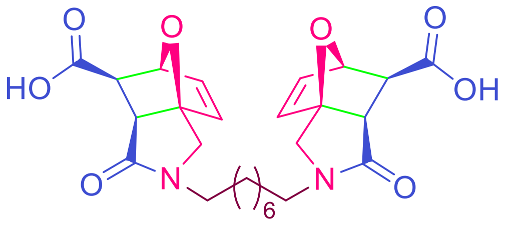

Orange solid, mp 66–67 °C; ^{1}H NMR (600 MHz, DMSO-d$_6$) δ 6.54 (*d*, *J* = 5.7 Hz, 2H, H$_g$), 6.39 (*dd*, J_1 = 5.7 Hz, J_2 = 1.8 Hz, 2H, H$_f$), 4.93 (*d*, *J* = 1.7 Hz, 2H, H$_c$), 3.99 (*d*, *J* = 11.7 Hz, 2H, H$_d$), 3.95 (*d*, *J* = 11.9 Hz, 2H, H$_e$), 3.51 (*d*, *J* = 11.7 Hz, 2H, H$_h$), 3.25-3.20 (*m*, 2H, H$_i$), 3.07-3.02 (*m*, 2H, H$_a$), 2.72 (*d*, *J* = 9.1 Hz, 2H, H$_b$), 1.43-1.39 (*m*, 4H, 2CH$_2$), 1.27-1.18 (*m*, 8H, 4CH$_2$); ^{13}C NMR (150 MHz, DMSO-d$_6$) δ 173.35, 170.60, 137.10, 136.09, 88.80, 81.52, 50.66, 48.03, 44.93, 42.07, 29.00, 27.07, 26.36; Anal calc for $C_{26}H_{32}N_2O_8$ (500.55): C 62.39, H 6.44, N 5.60. Found: C 62.46, H 6.50, N 5.67.

7-Carboxy-1-oxo-1,6,7,7a-tetrahydro-3a,6-epoxyisoindol-2(3H)-yl)dodecyl)-1-oxo-1,2,3,6,7,7a-hexahydro-3a,6-epoxyisoindole-7-carboxylic acid ***(2p)***

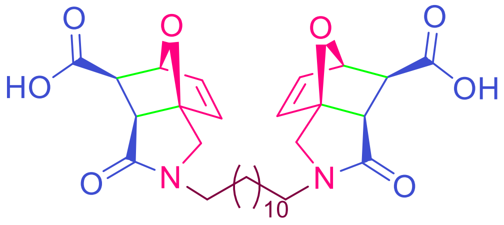

Beige solid, mp 94–95 °C; ^{1}H NMR (600 MHz, $CDCl_3$) δ 6.49 (*d*, *J* = 5.7 Hz, 2H, H_g), 6.45 (*d*, *J* = 5.7 Hz, 2H, H_f), 5.24 (*d*, *J* = 2 Hz, 2H, H_c), 3.98 (*m*, 2H, H_d), 3.72 (*d*, *J* = 11.9 Hz, 2H, H_e), 3.41-3.32 (*m*, 2H, H_h), 3.28-3.19 (*m*, 2H, H_i), 2.89 (*d*, *J* = 9.1 Hz, 2H, H_a), 2.80-2.78 (*m*, 2H, H_b), 1.51 (*quin*, *J* = 7.02 Hz, 4H, $2CH_2$), 1.26-1.22 (*m*, 16H, $8CH_2$); ^{13}C NMR (150 MHz, $CDCl_3$) δ 173.46, 172.37, 137.17, 134.95, 88.91, 82.35, 50.76, 48.95, 45.85, 43.16, 29.31, 29.26, 29.05, 26.94, 26.48; Anal calc for $C_{30}H_{40}N_2O_8$ (556.66): C 64.73, H 7.24, N 5.03. Found: C 64.68, H 7.18, N 5.10.

^{1}H NMR spectrum of compound **2a**

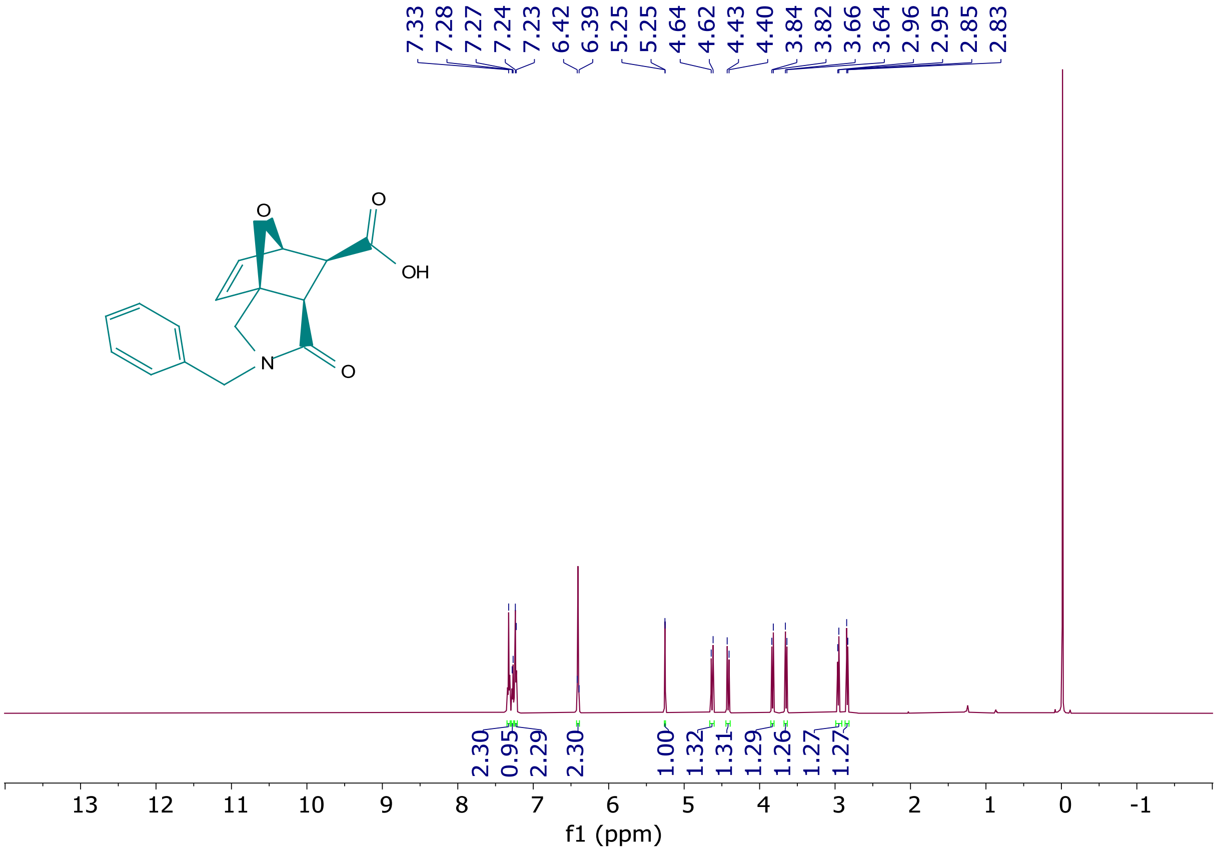

^{1}H NMR spectrum of compound **2b**

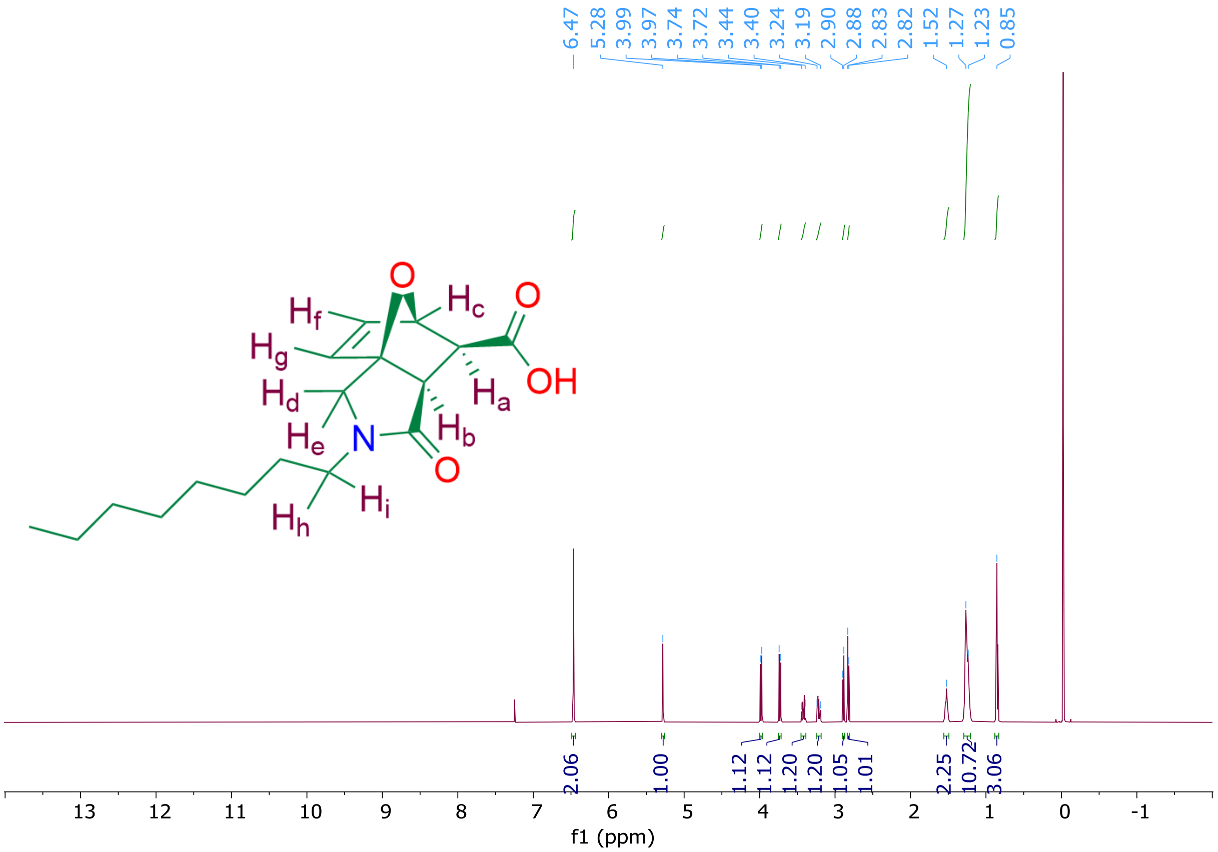

^{1}H NMR spectrum of compound **2c**

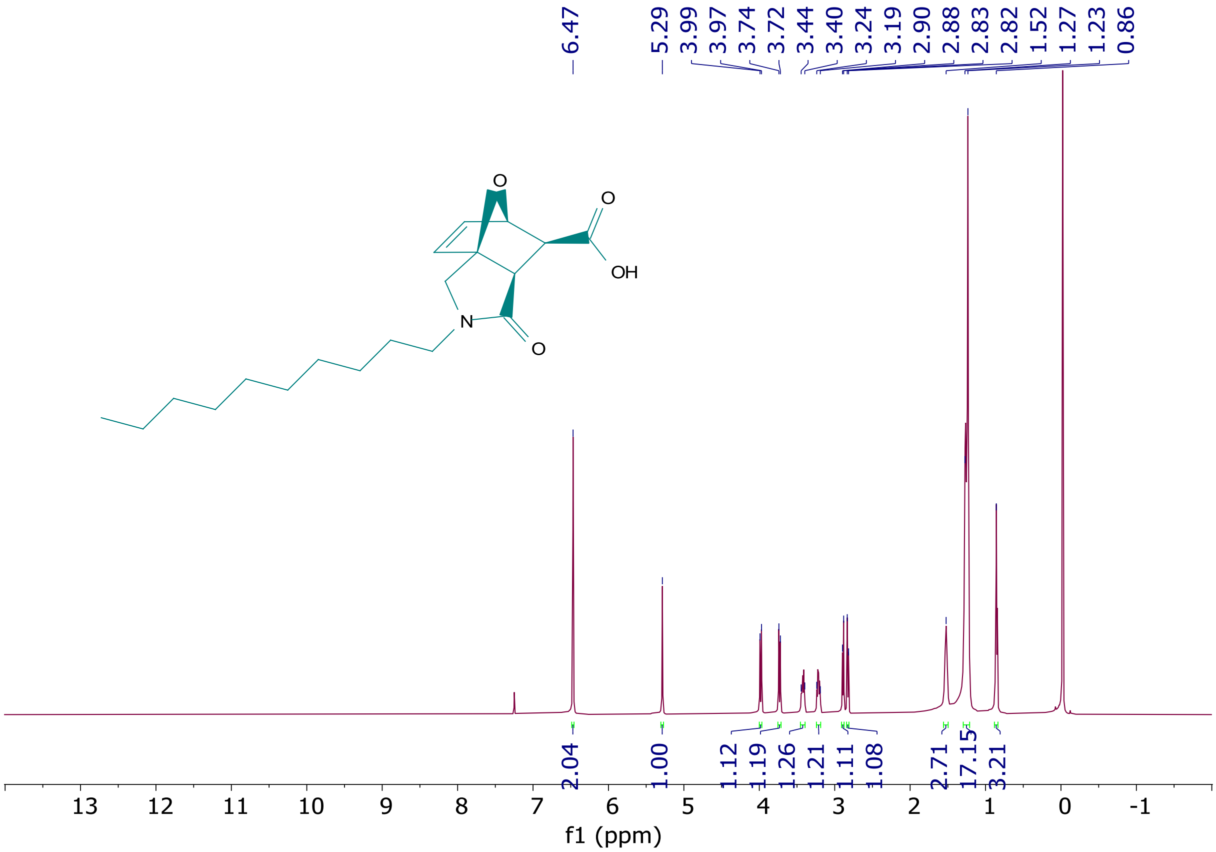

^{1}H NMR spectrum of compound **2d**

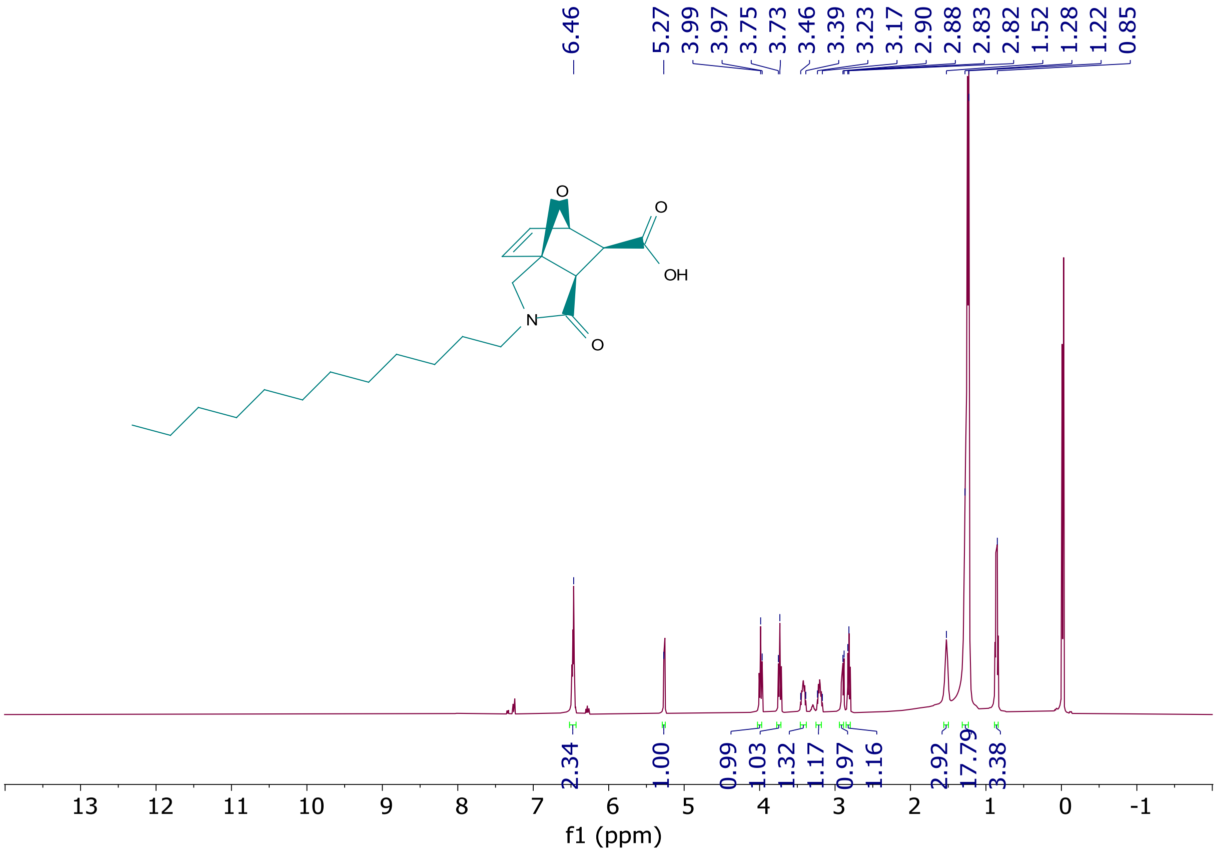

^{1}H NMR spectrum of compound **2e**

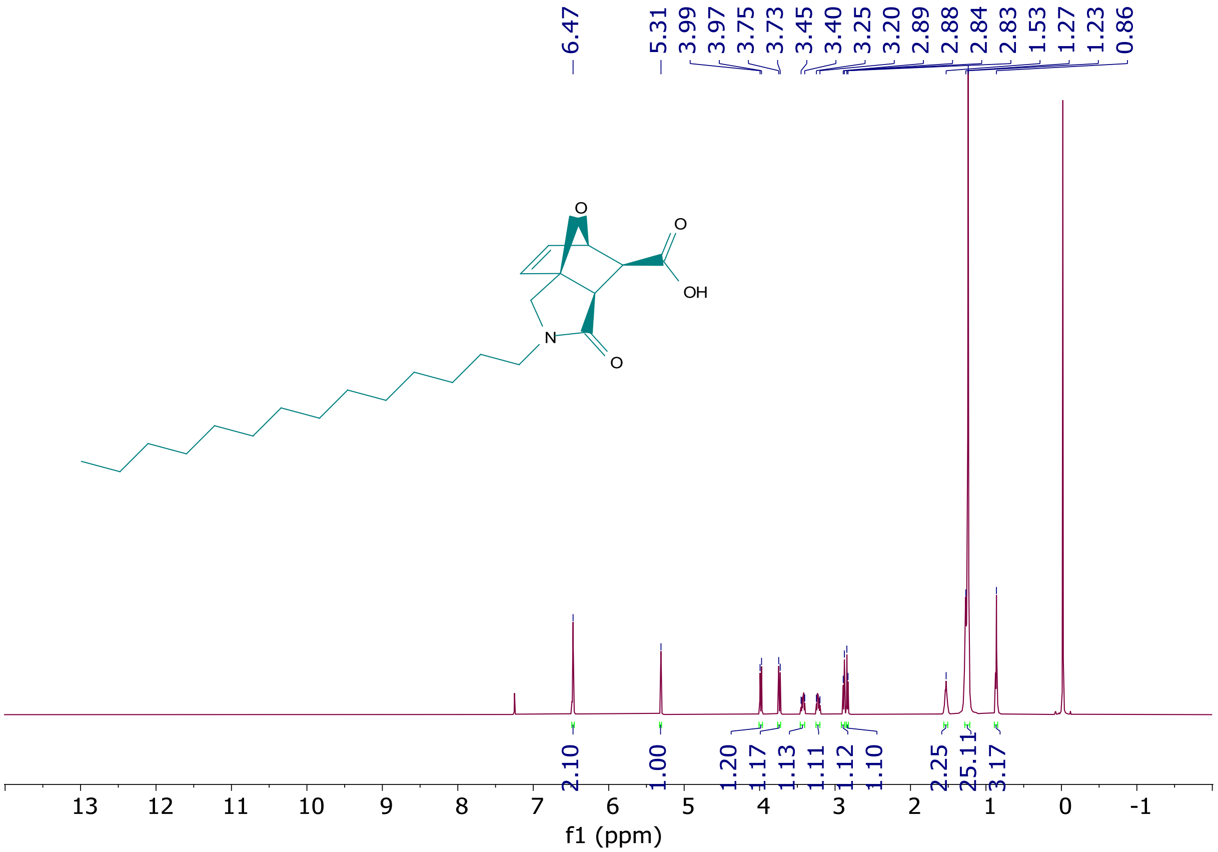

^{1}H NMR spectrum of compound **2f**

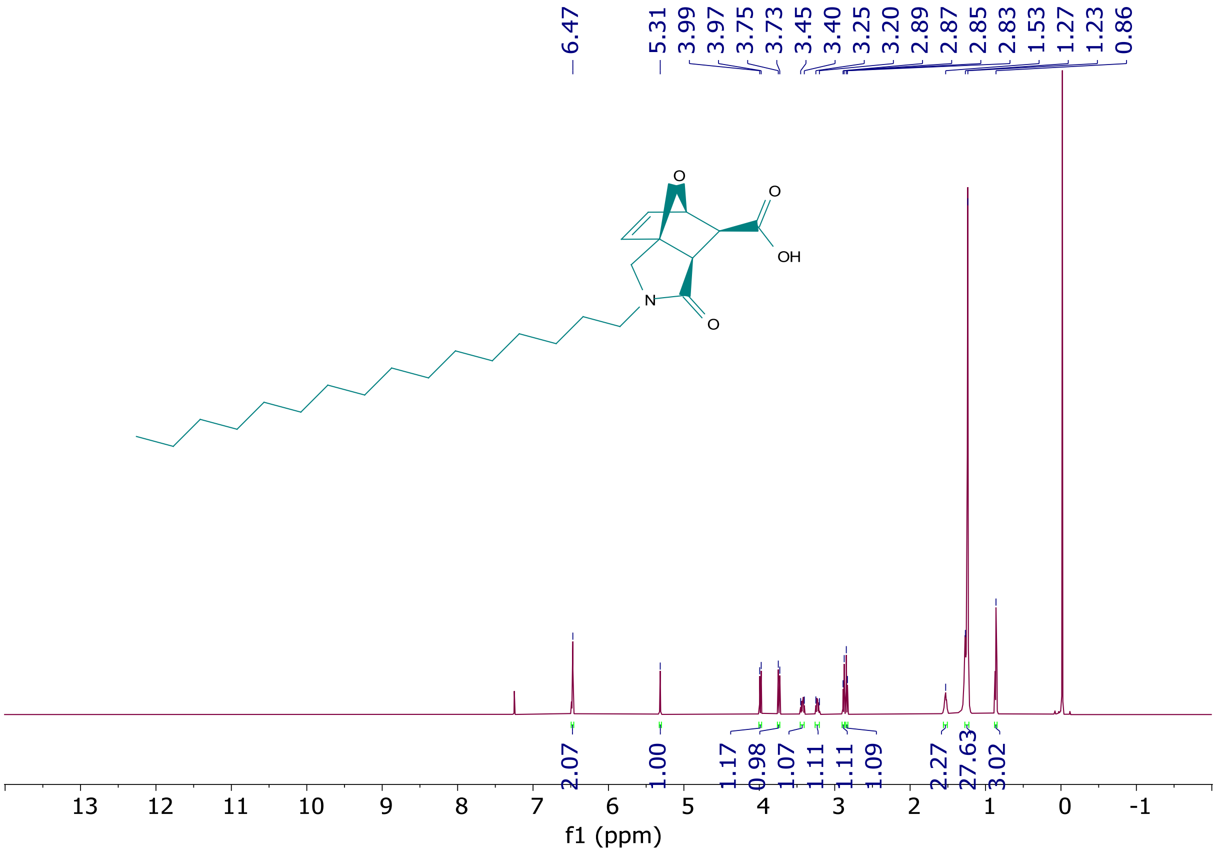

^{1}H NMR spectrum of compound **2g**

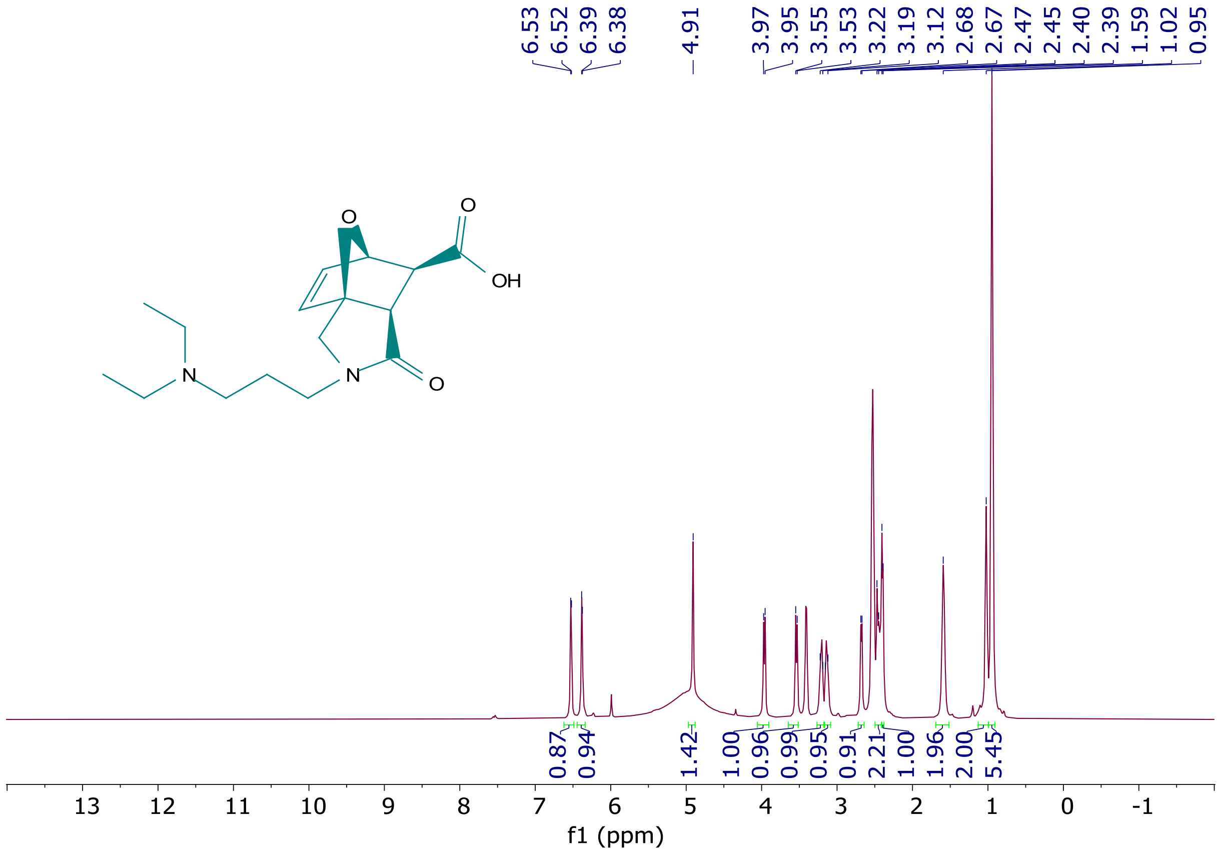

^{1}H NMR spectrum of compound **2h**

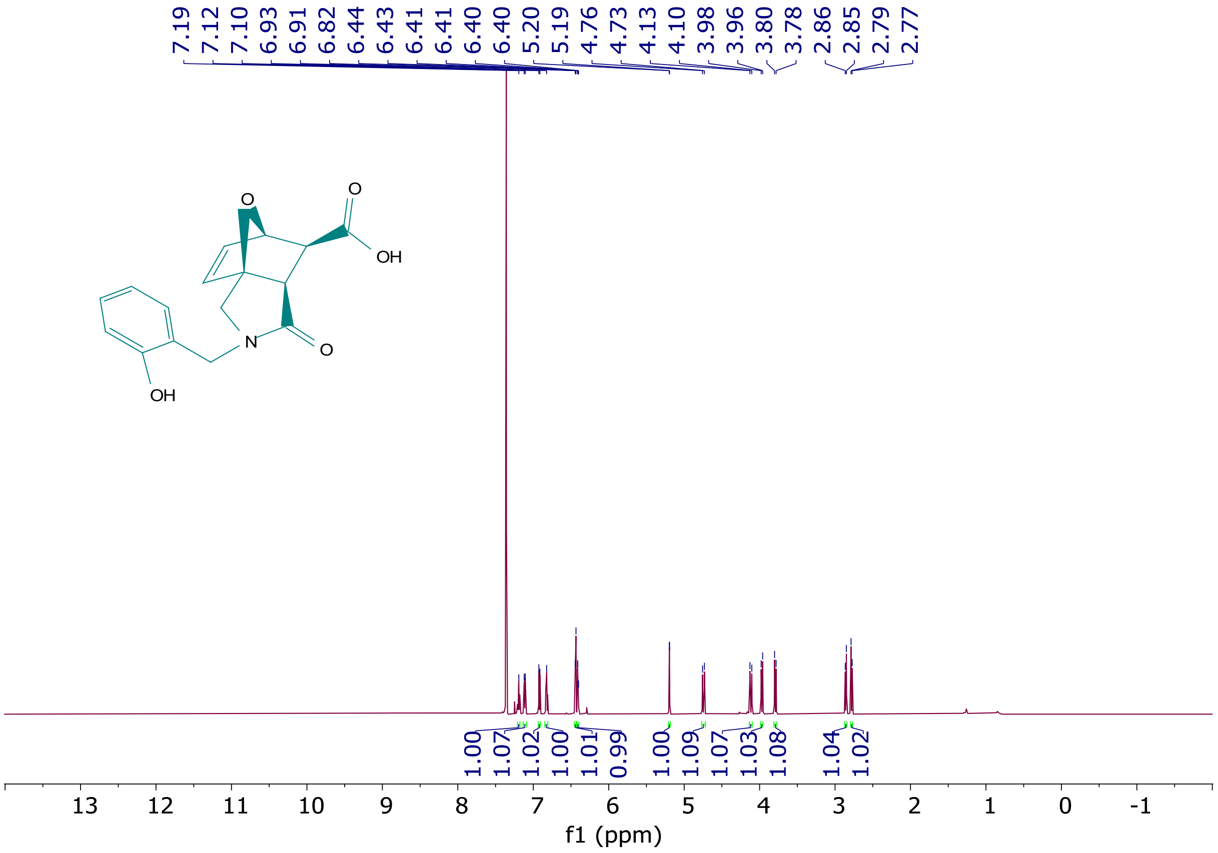

^{1}H NMR spectrum of compound **2i**

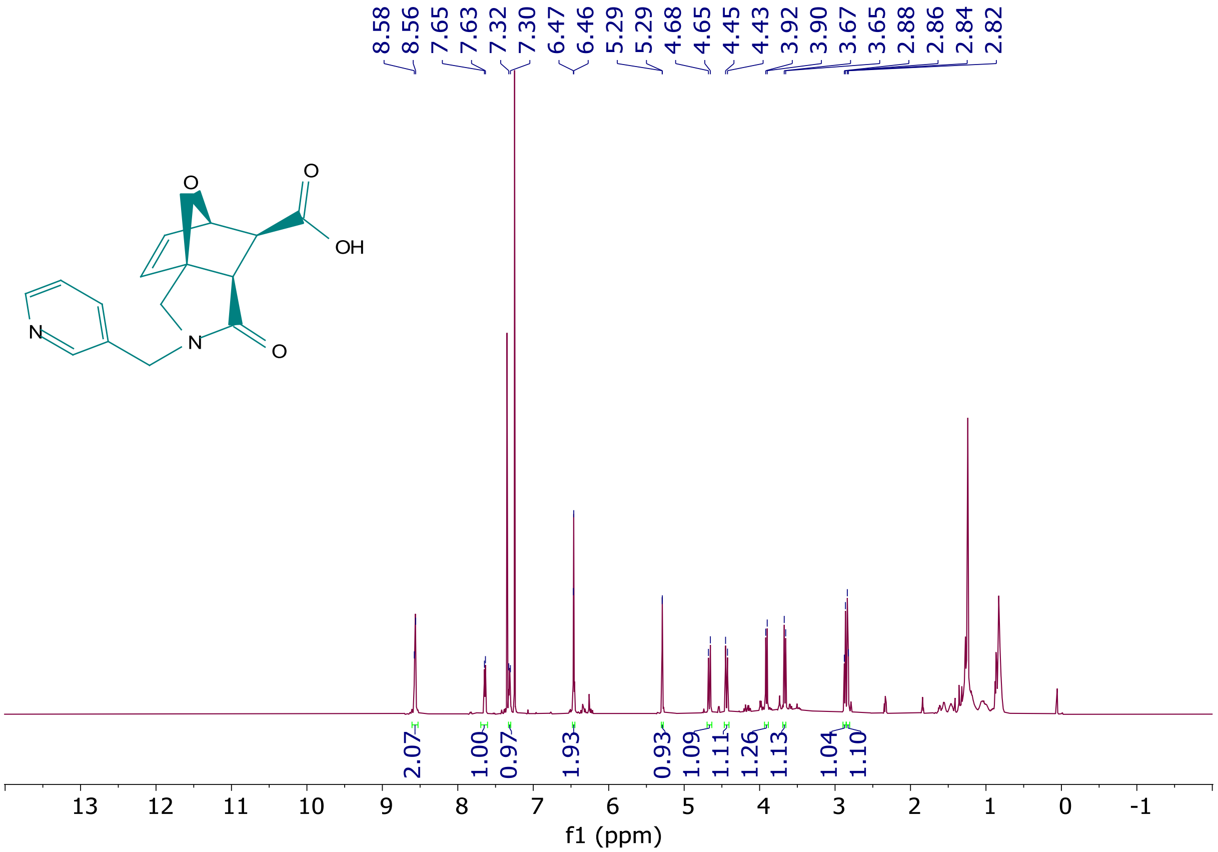

^{1}H NMR spectrum of compound **2j**

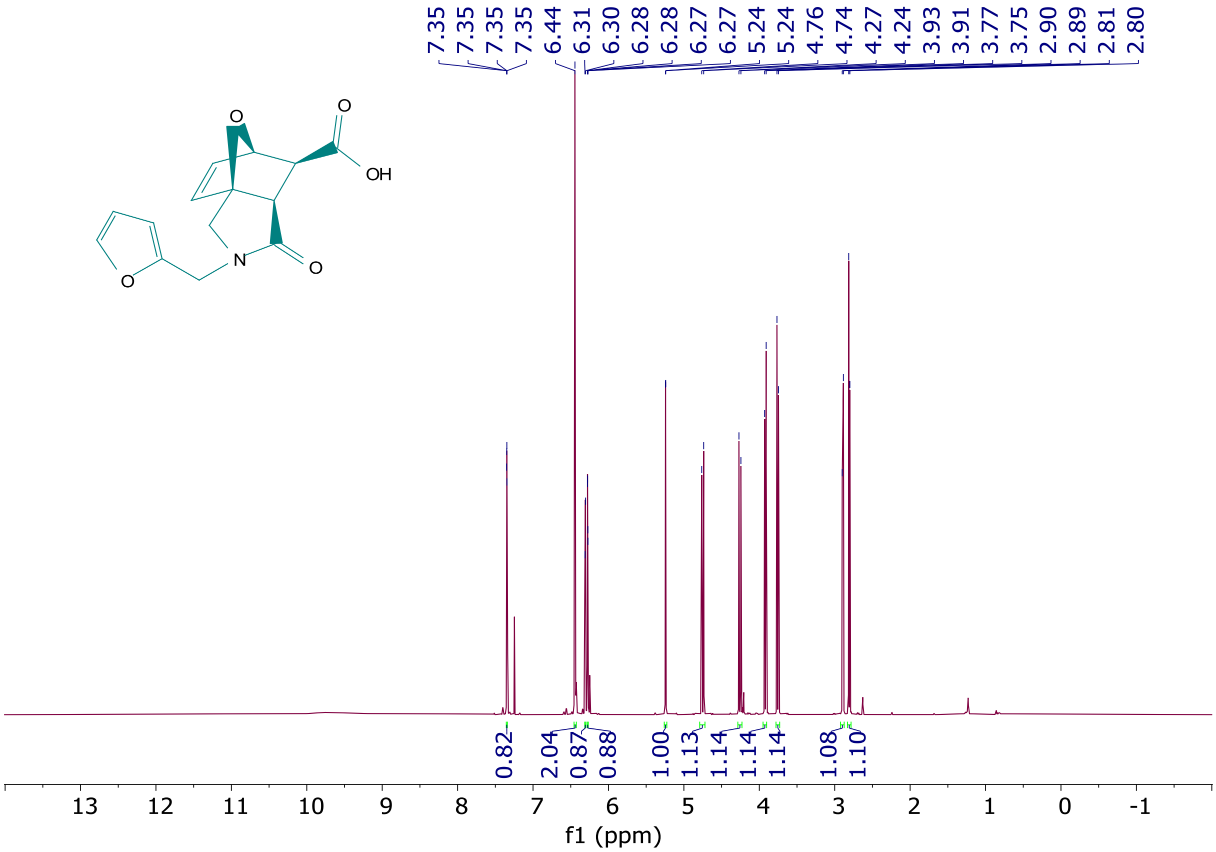

^{1}H NMR spectrum of compound **2k**

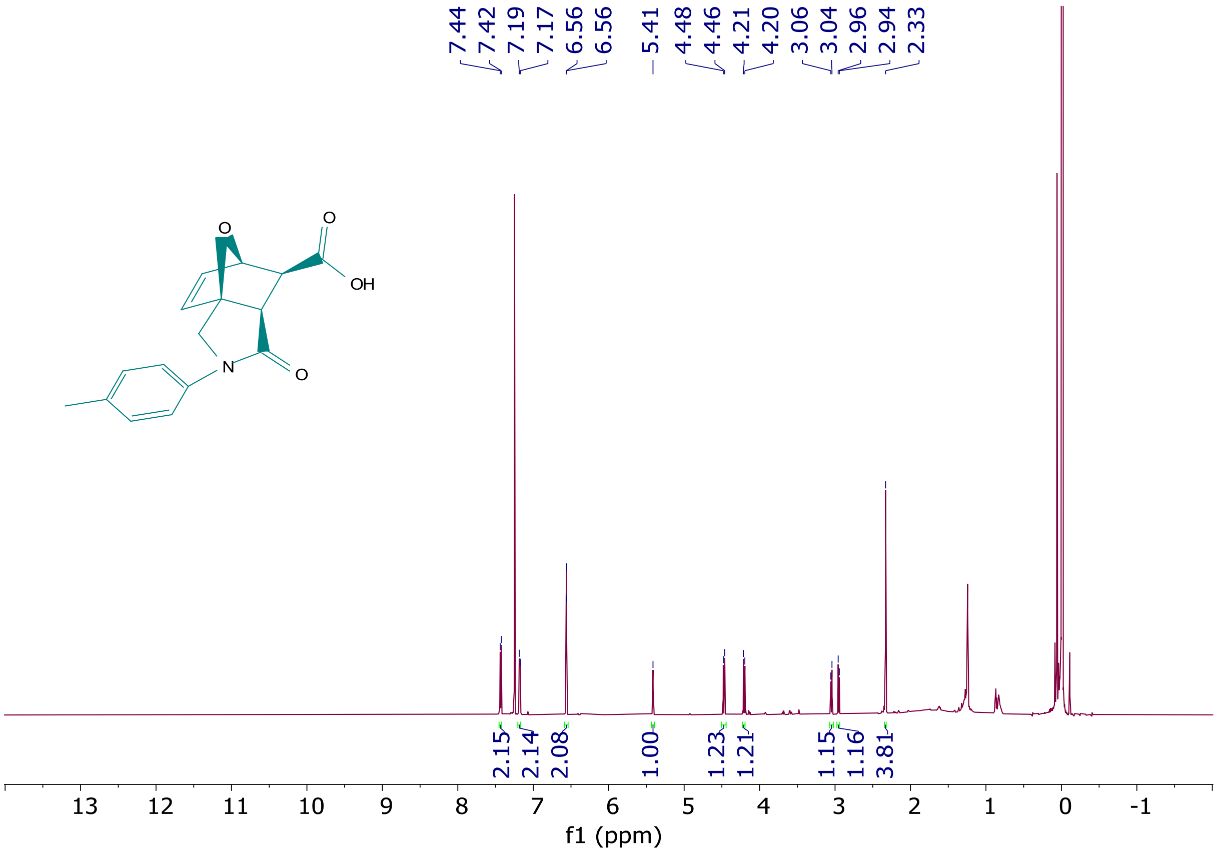

^{1}H NMR spectrum of compound **2l**

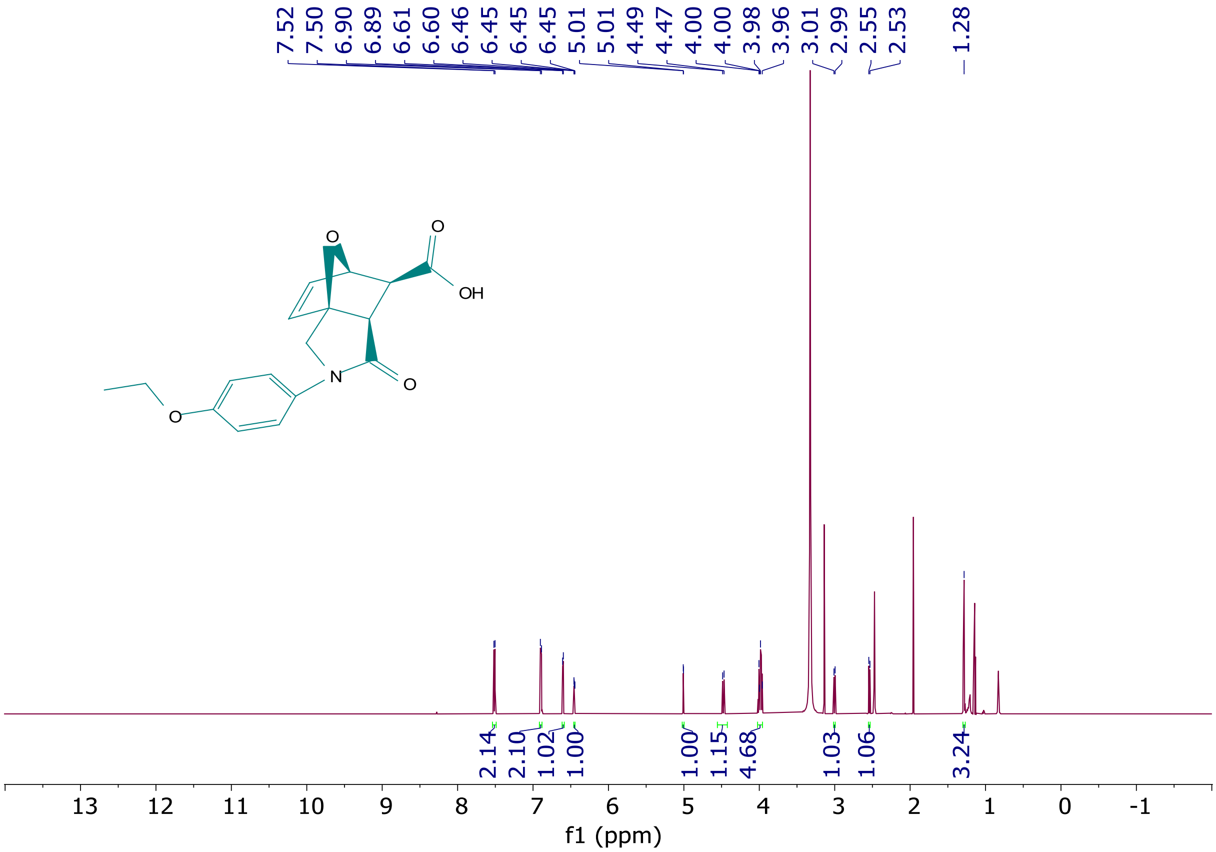

^{1}H NMR spectrum of compound **2m**

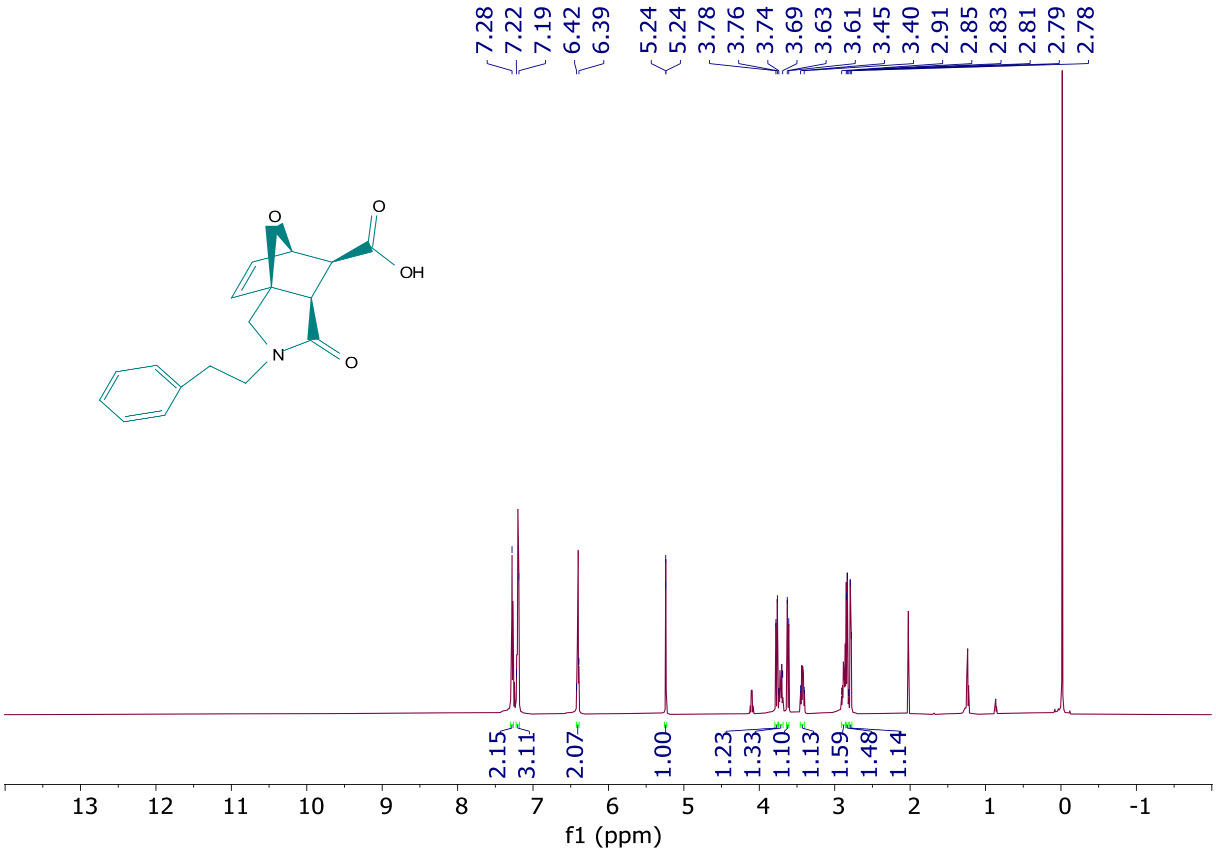

^{1}H NMR spectrum of compound **2n**

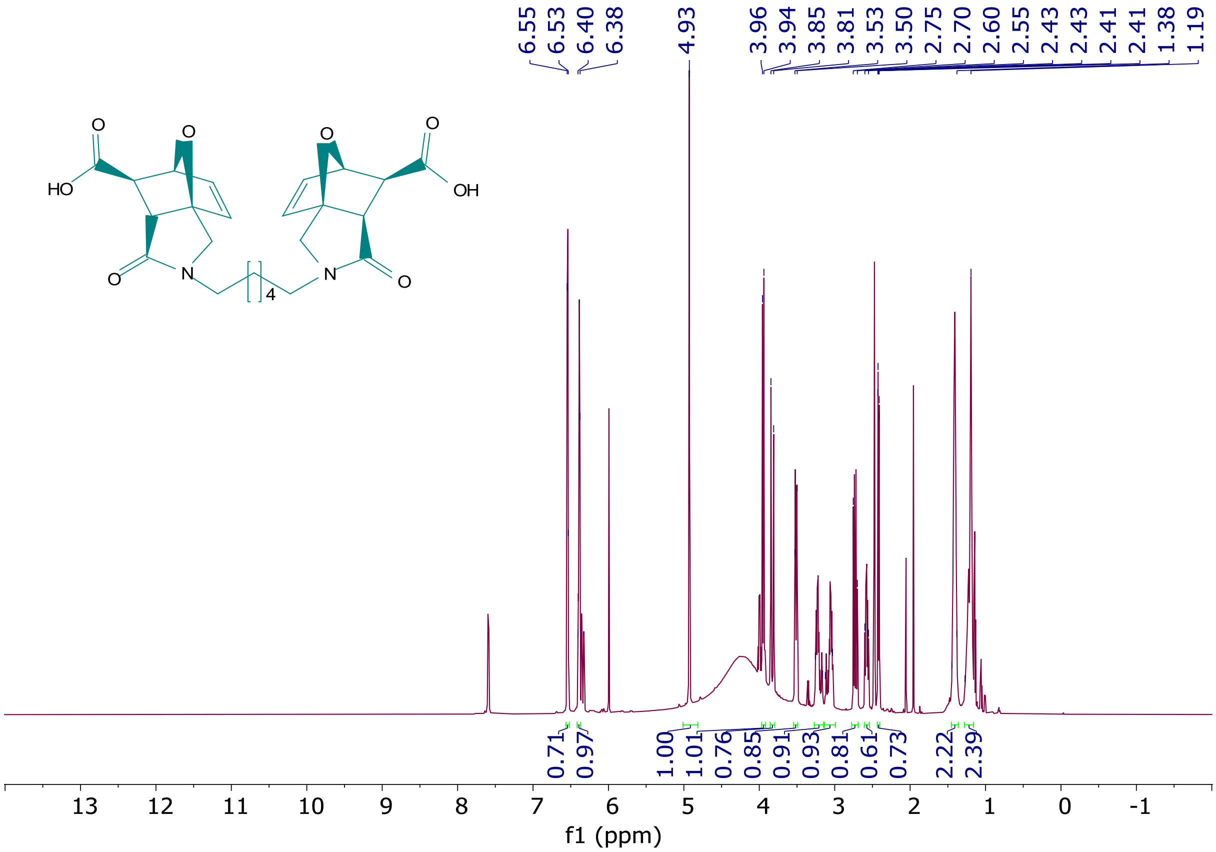

^{1}H NMR spectrum of compound **2o**

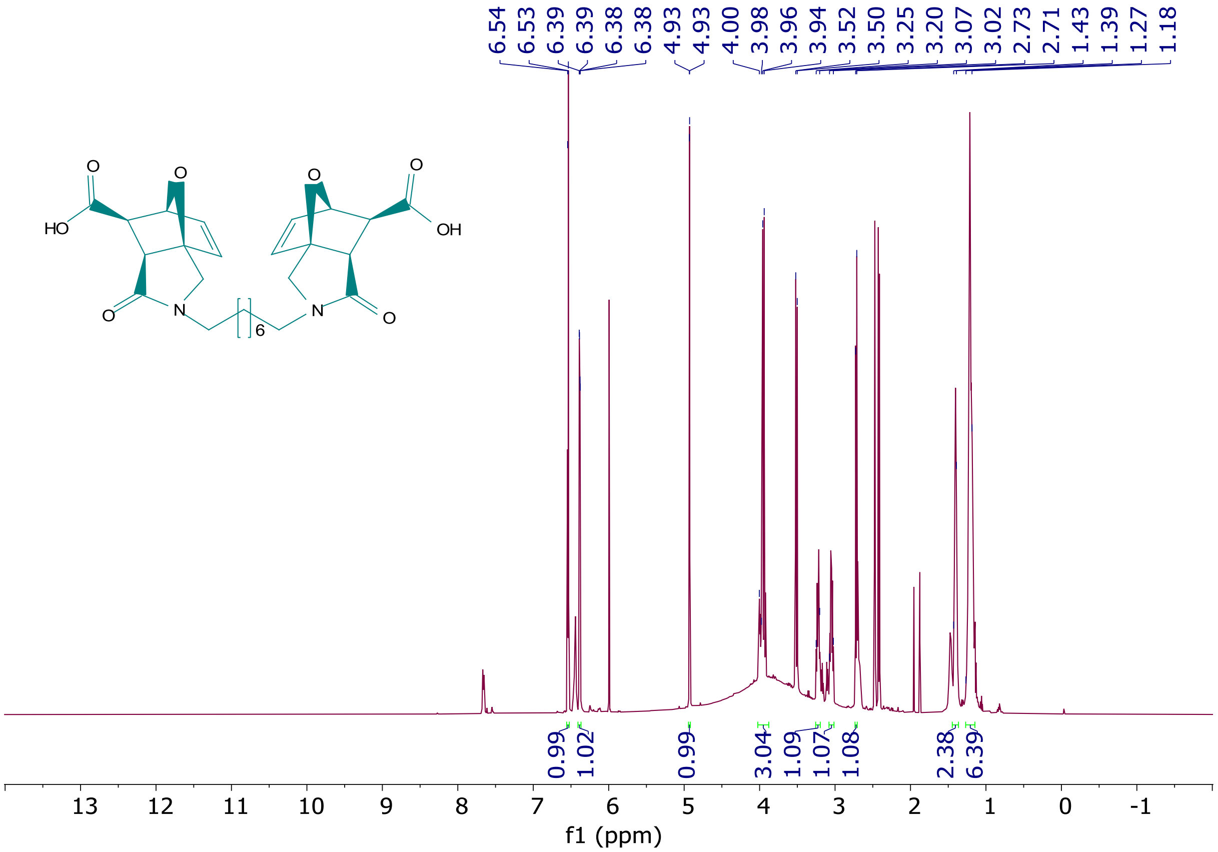

^{1}H NMR spectrum of compound **2p**

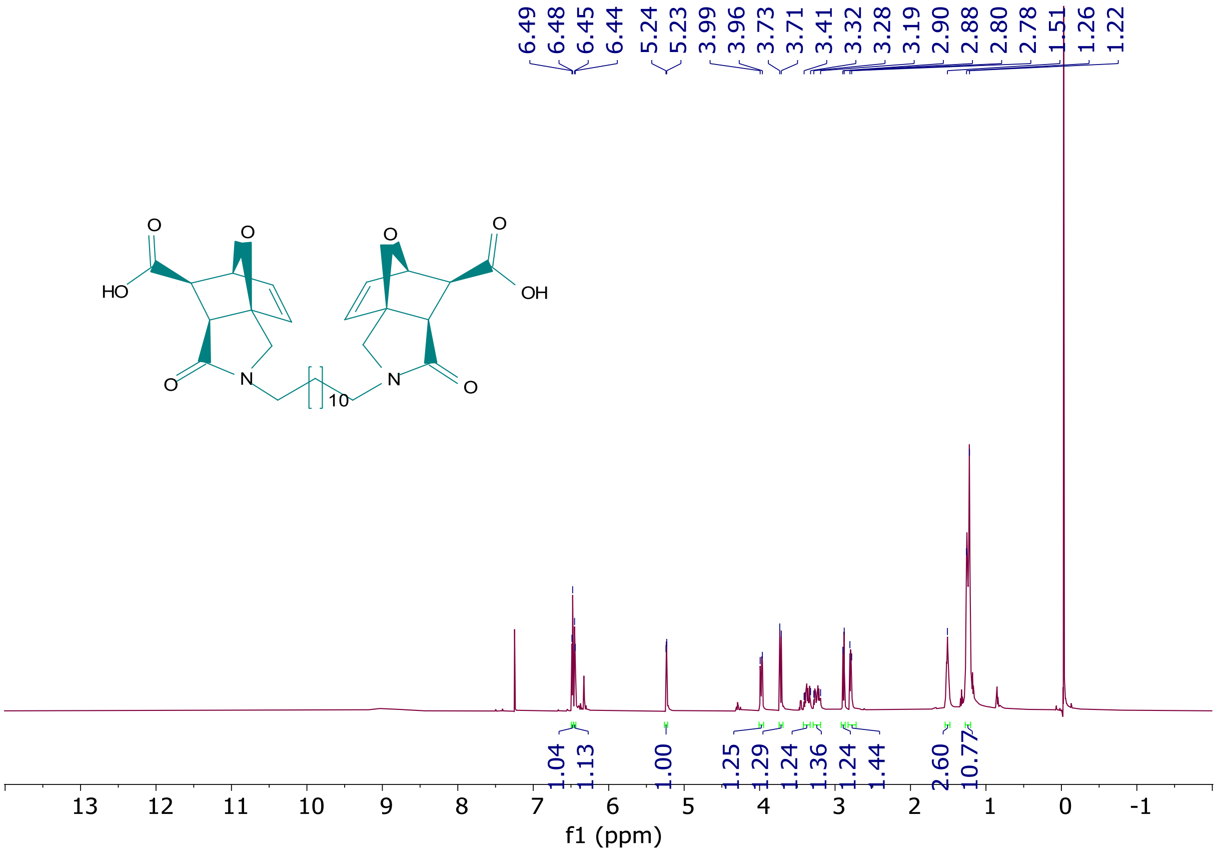

^{13}C NMR spectrum of compound **2a**

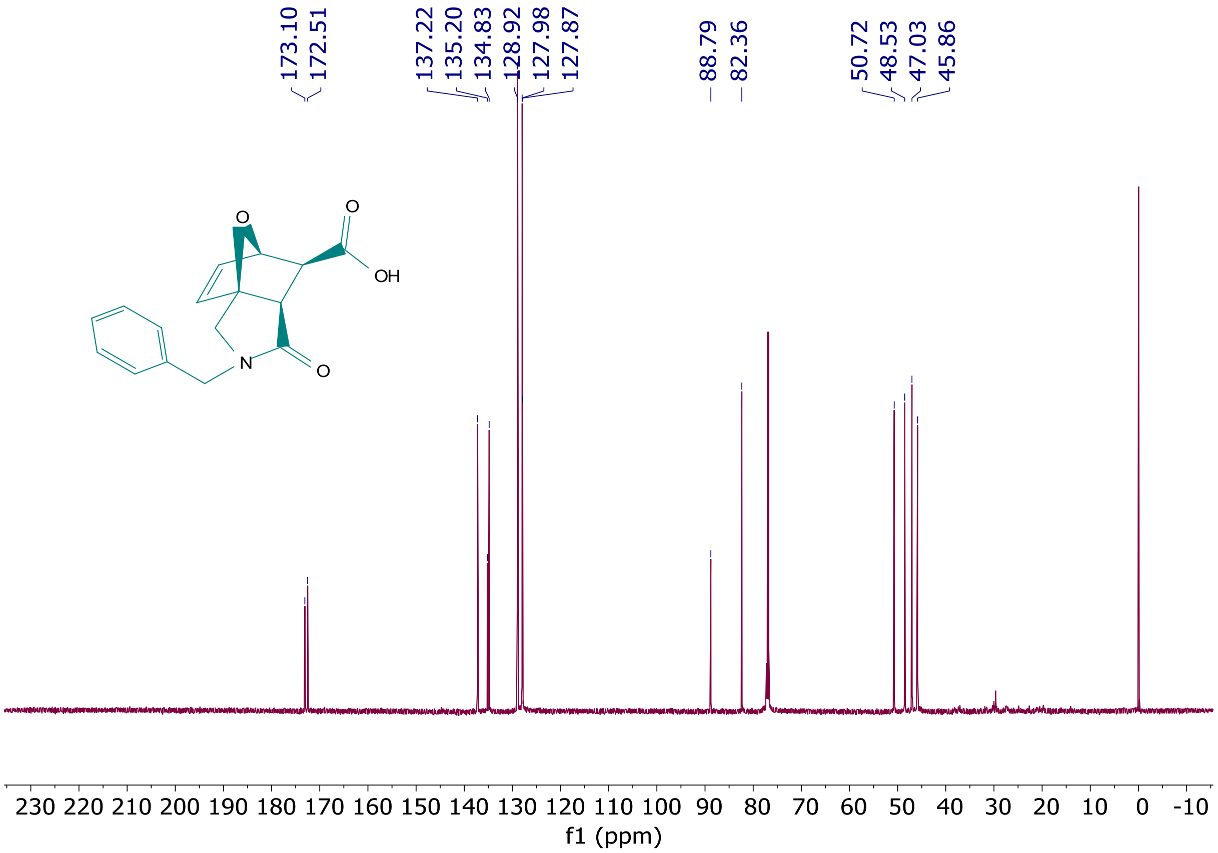

^{13}C NMR spectrum of compound **2b**

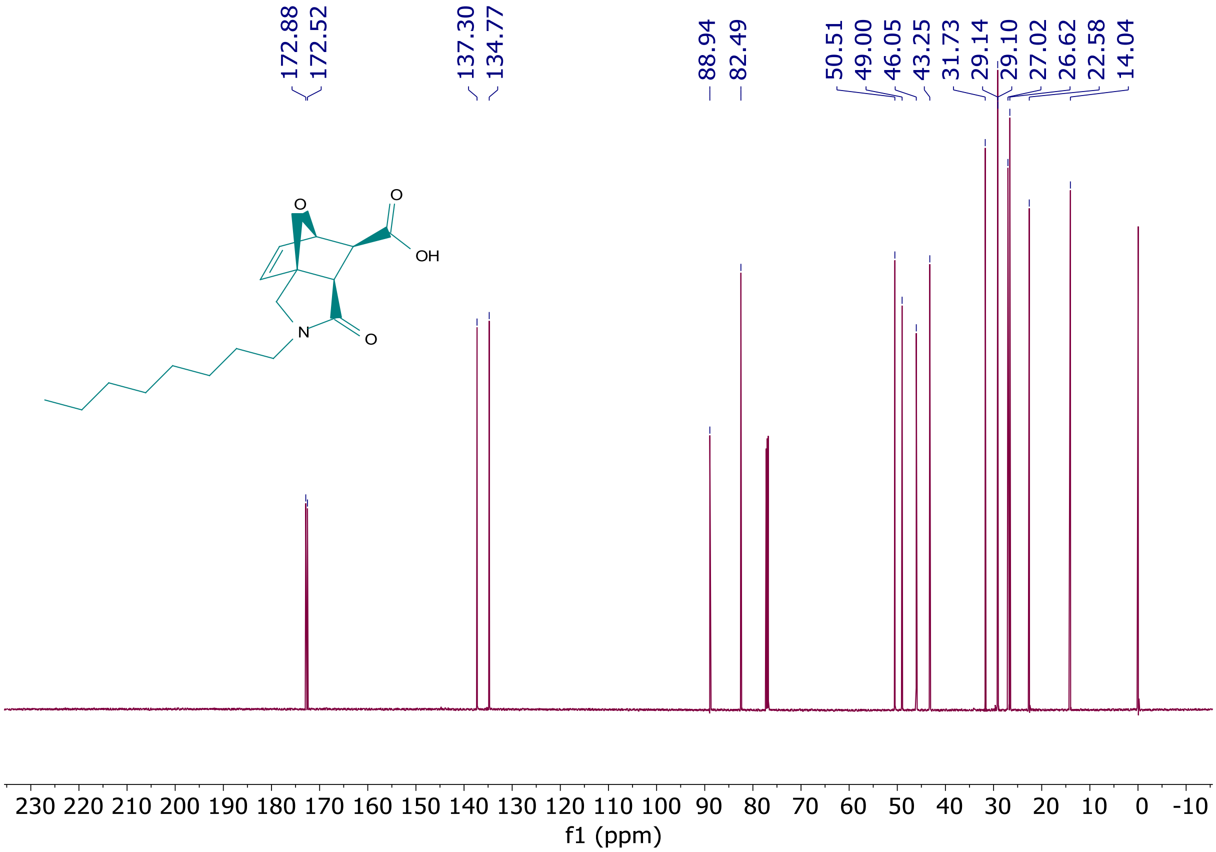

^{13}C NMR spectrum of compound **2c**

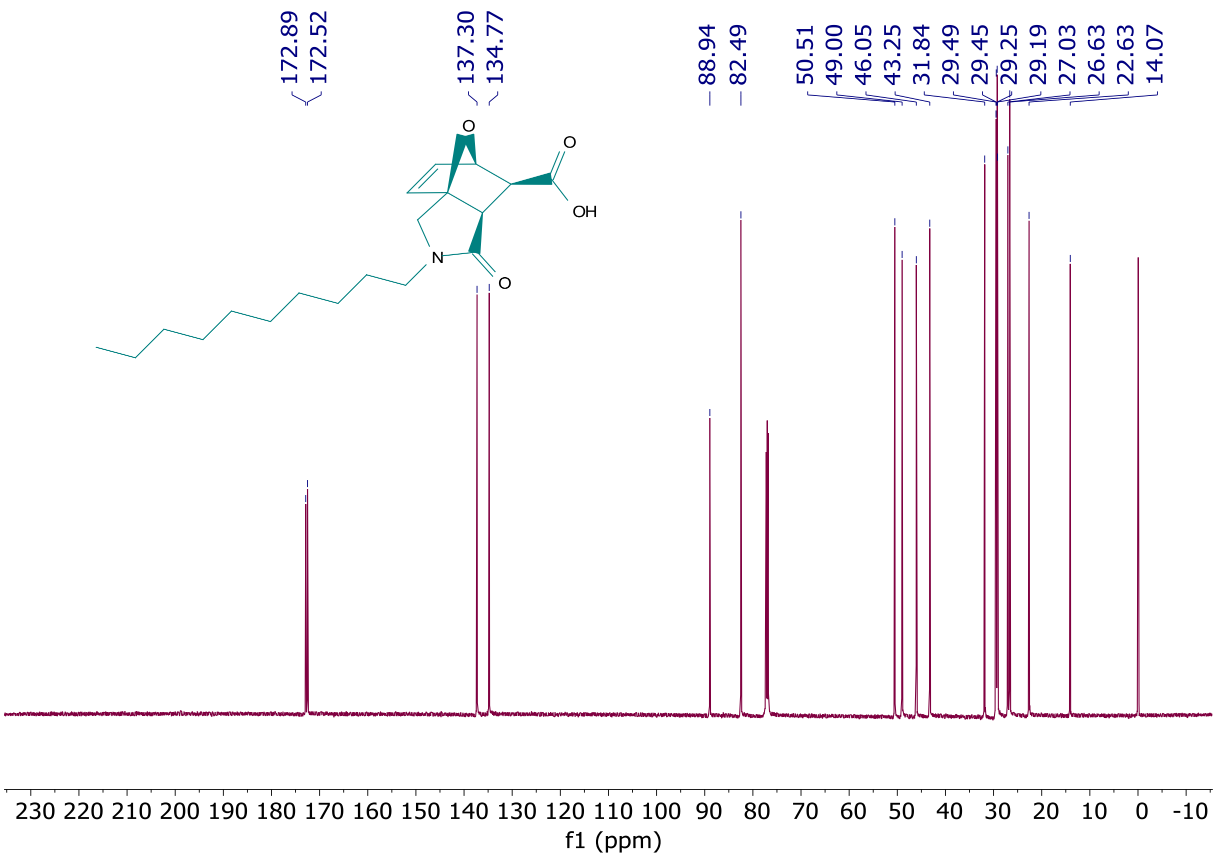

^{13}C NMR spectrum of compound **2d**

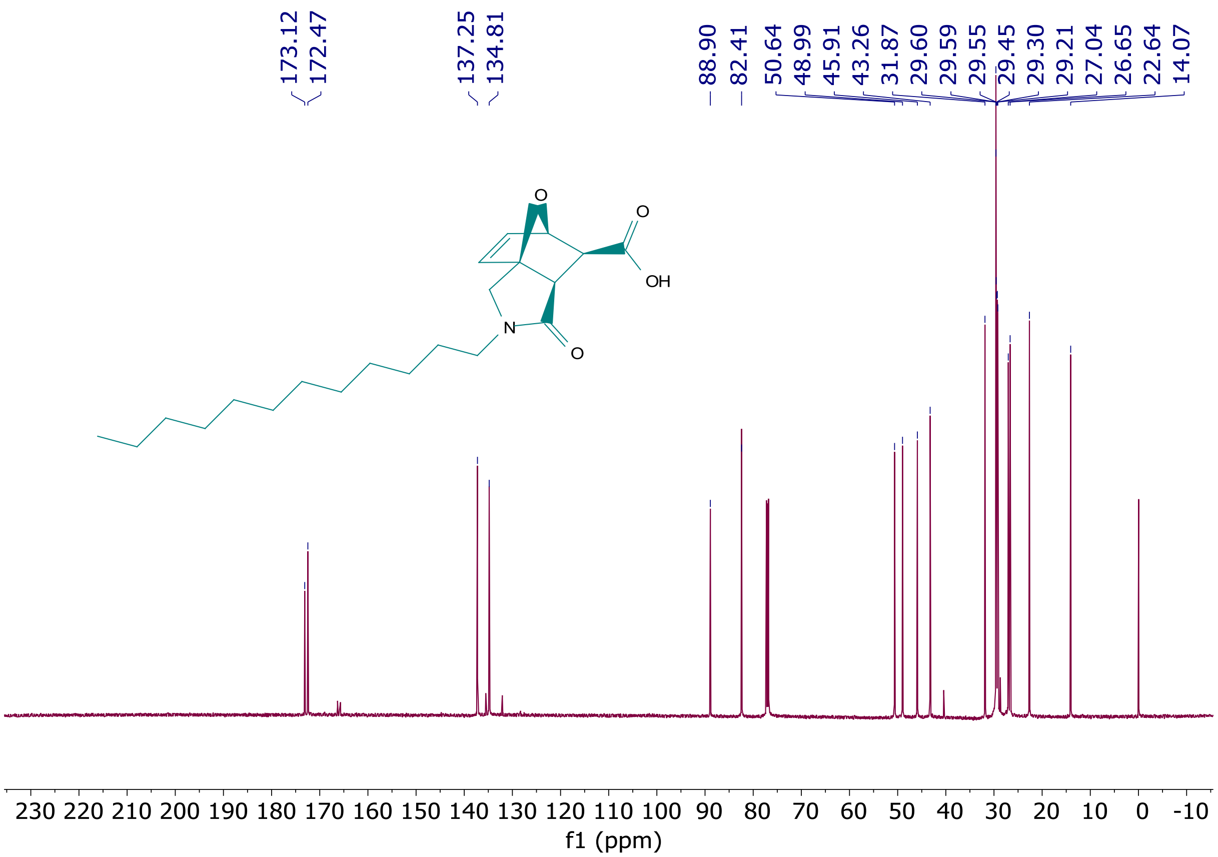

^{13}C NMR spectrum of compound **2e**

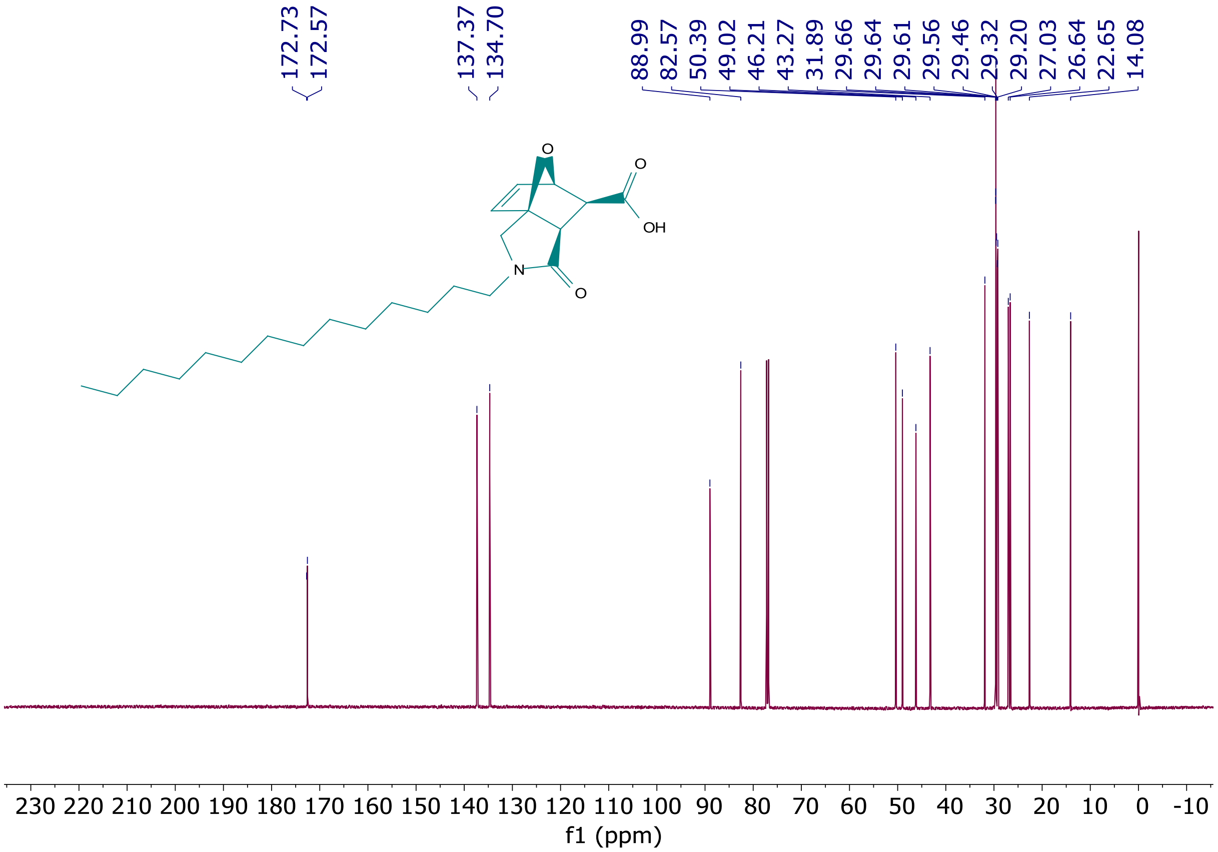

^{13}C NMR spectrum of compound **2f**

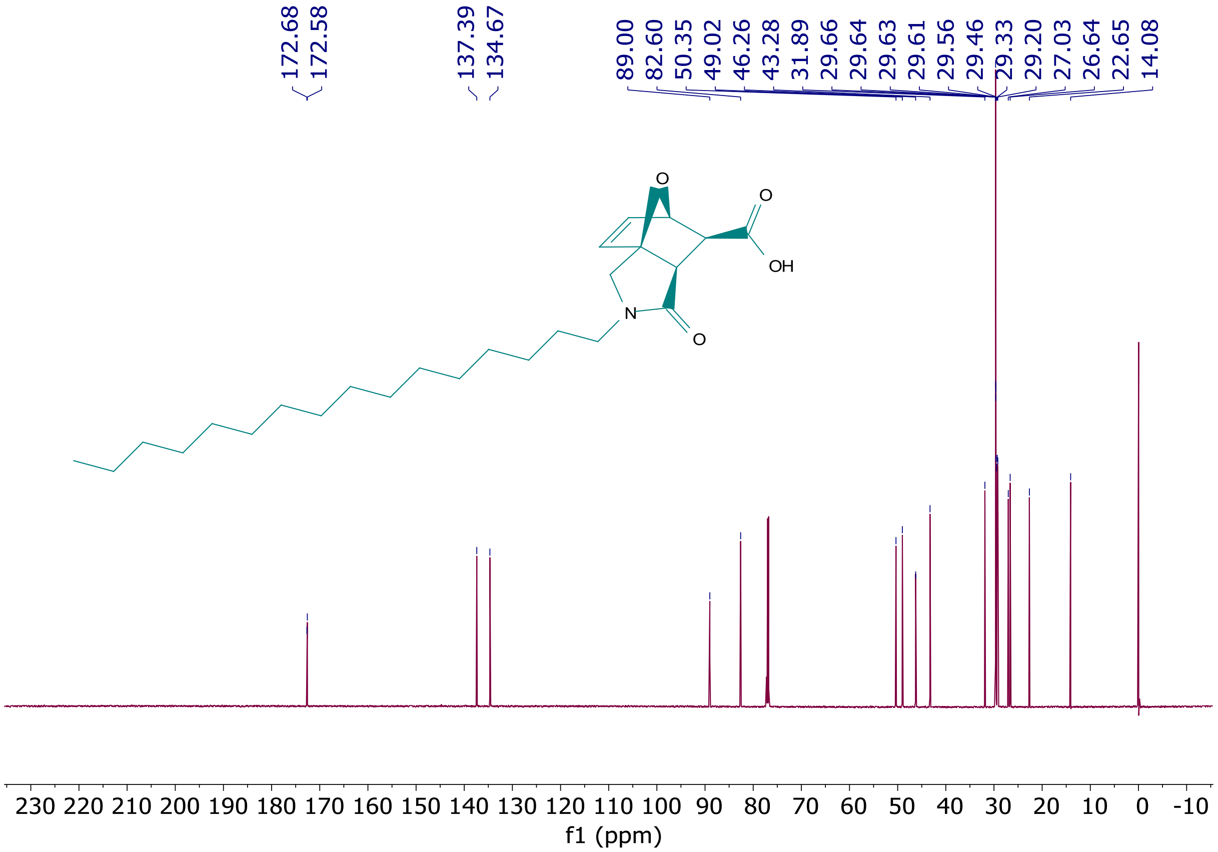

^{13}C NMR spectrum of compound **2g**

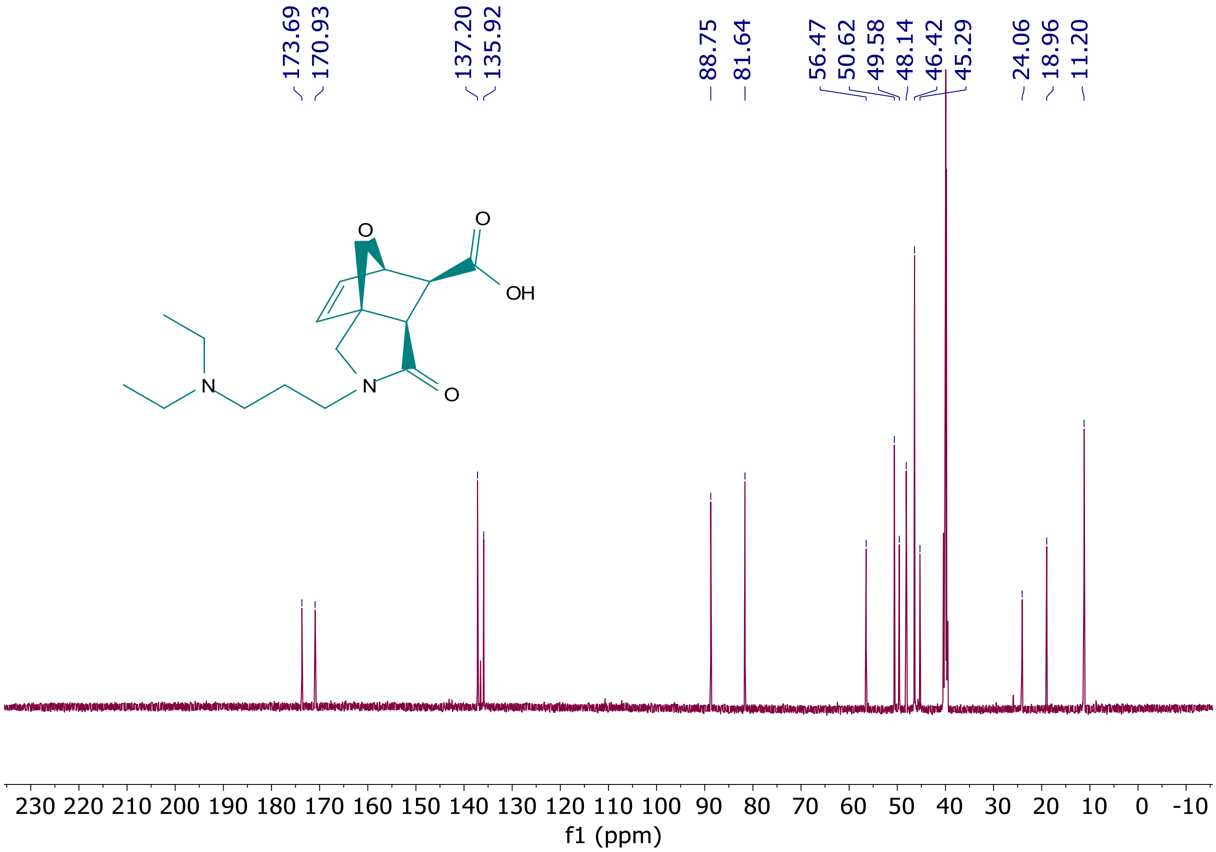

^{13}C NMR spectrum of compound **2h**

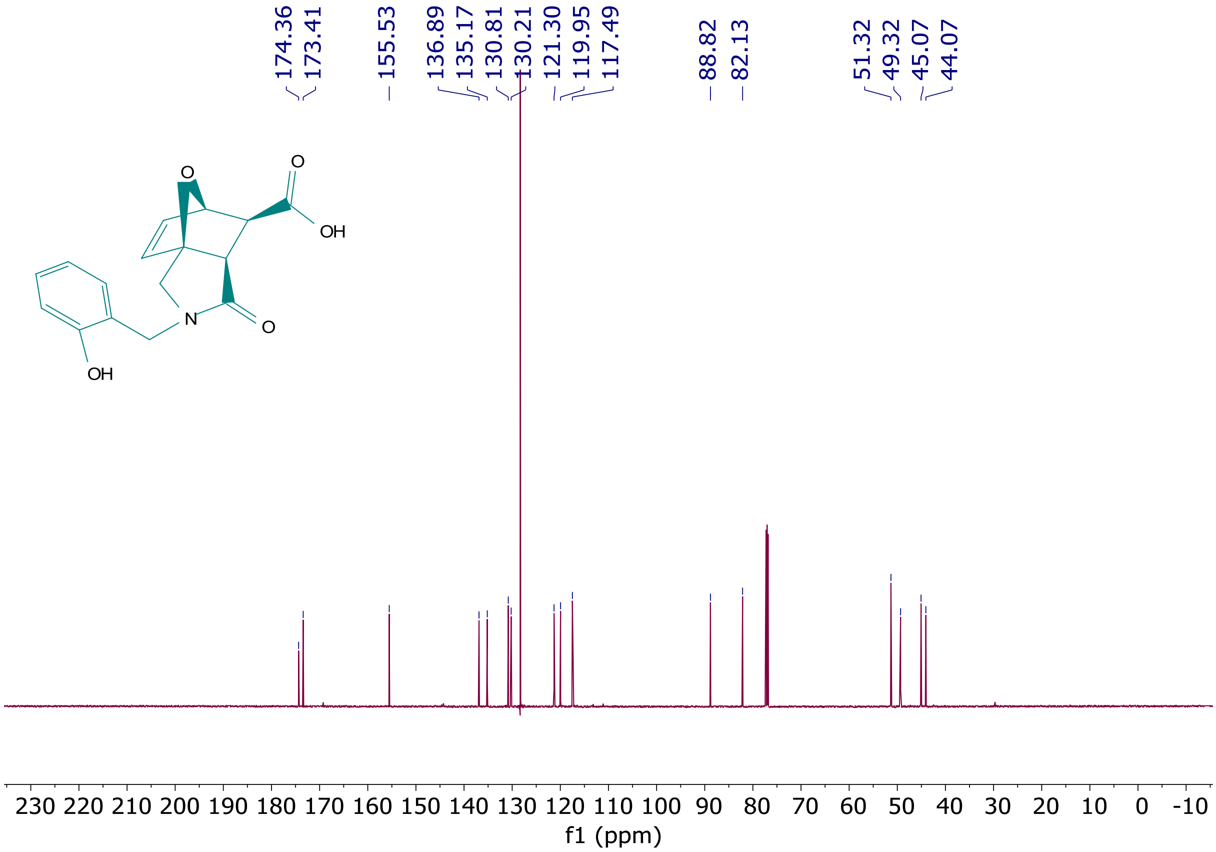

^{13}C NMR spectrum of compound **2i**

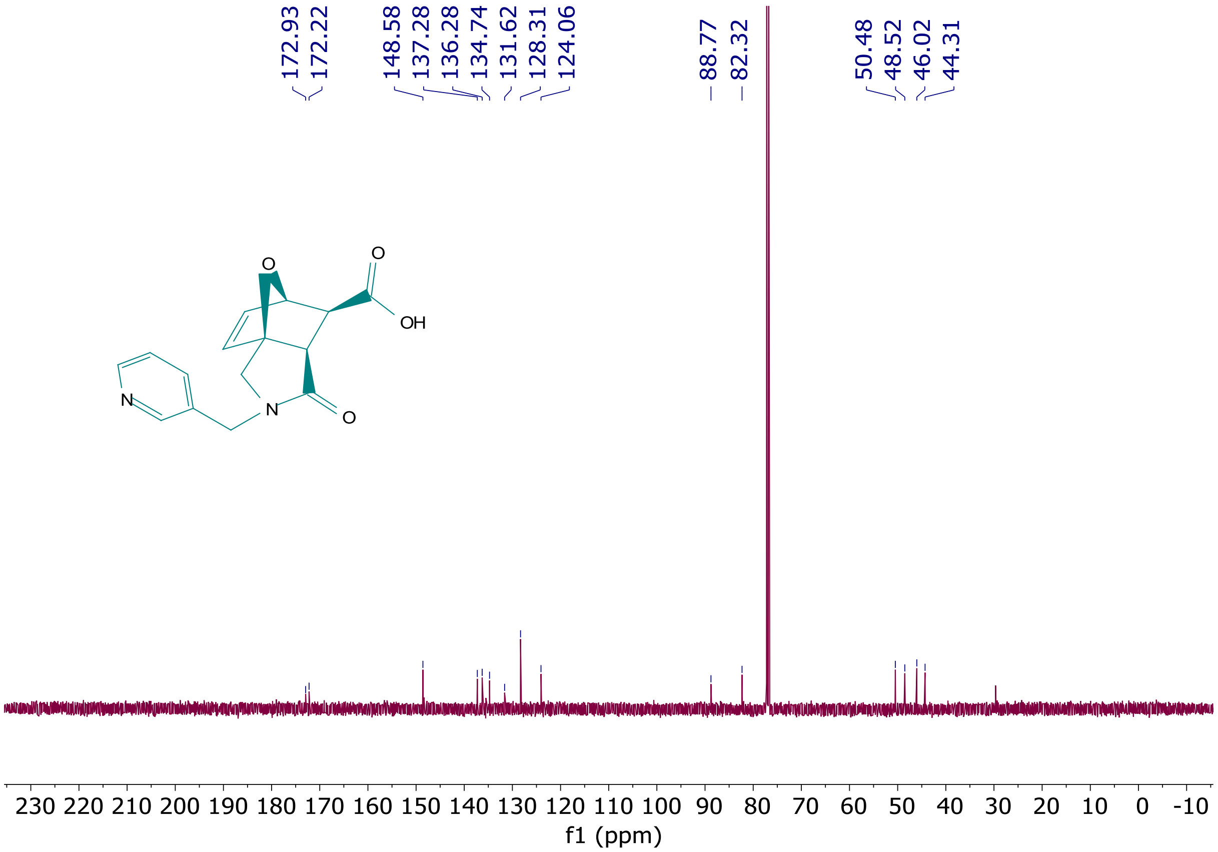

^{13}C NMR spectrum of compound **2j**

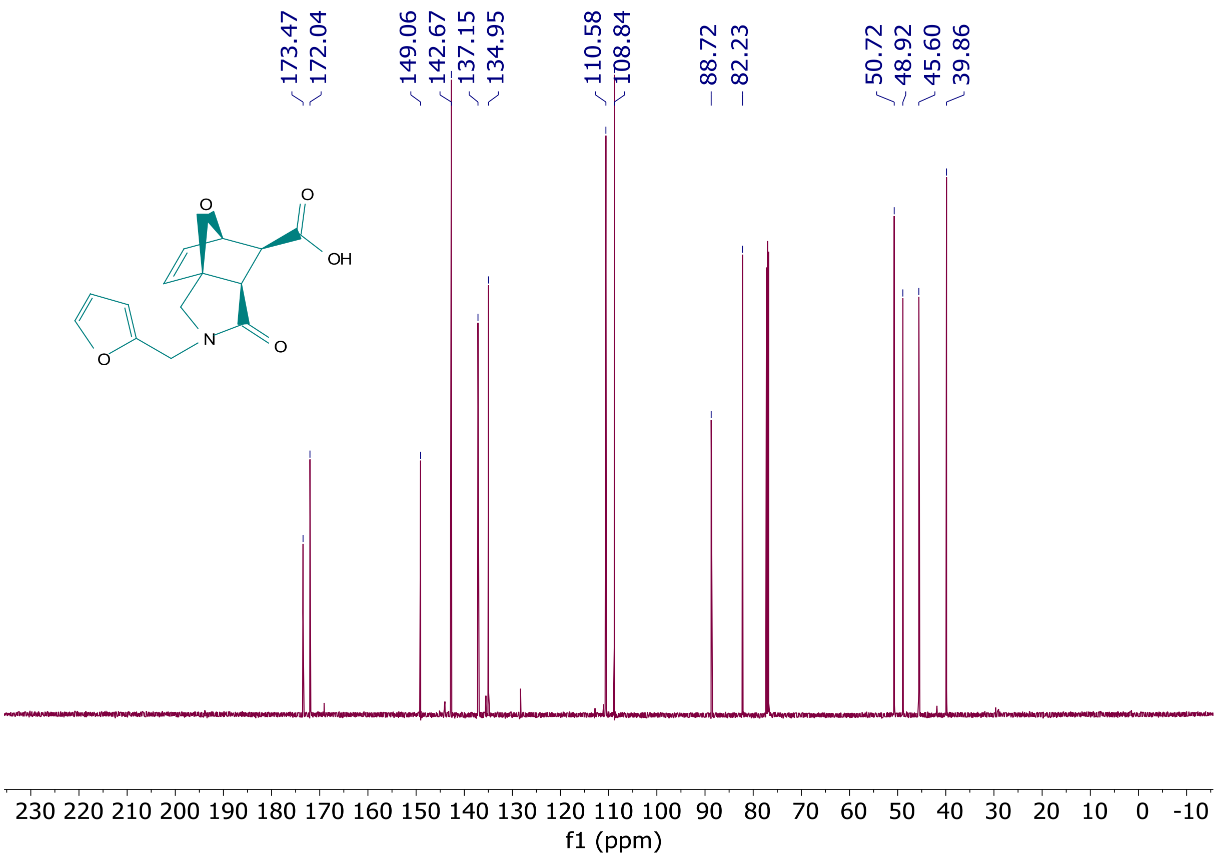

^{13}C NMR spectrum of compound **2k**

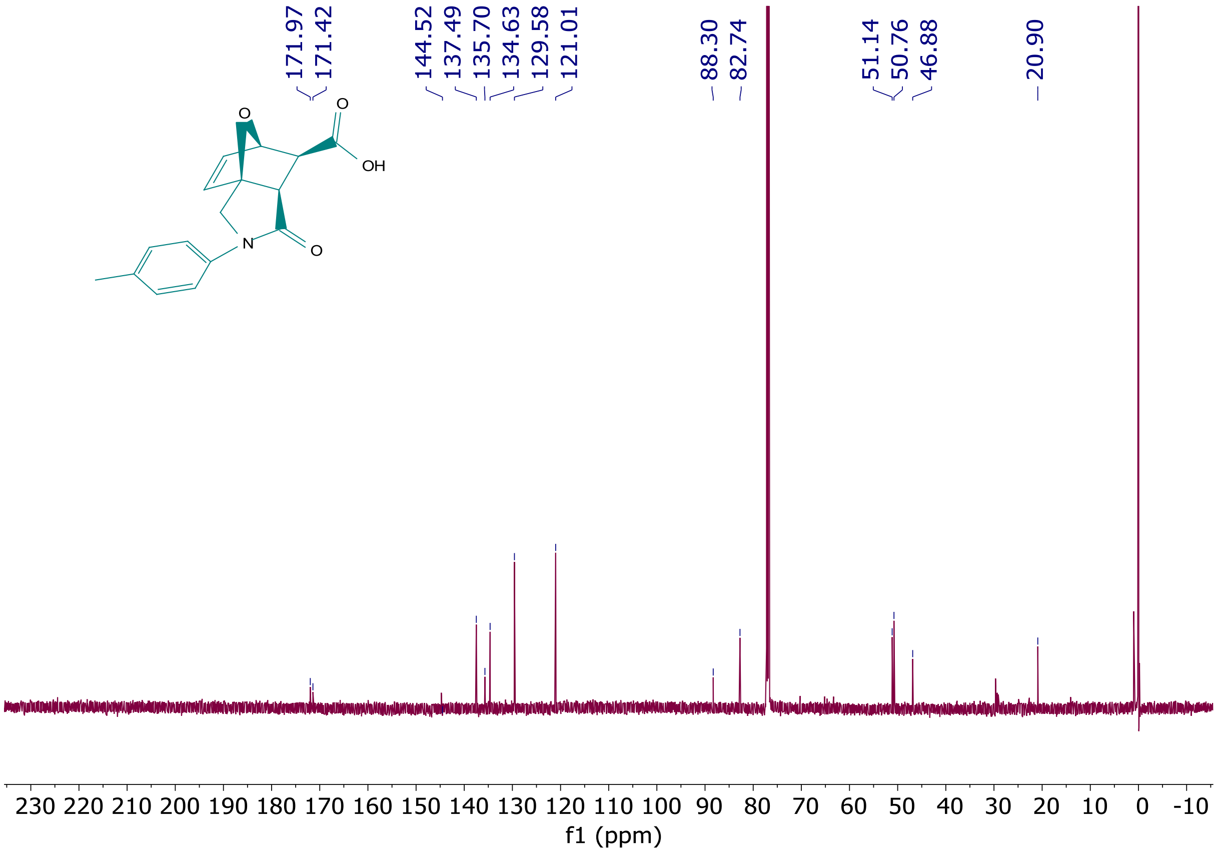

^{13}C NMR spectrum of compound **2l**

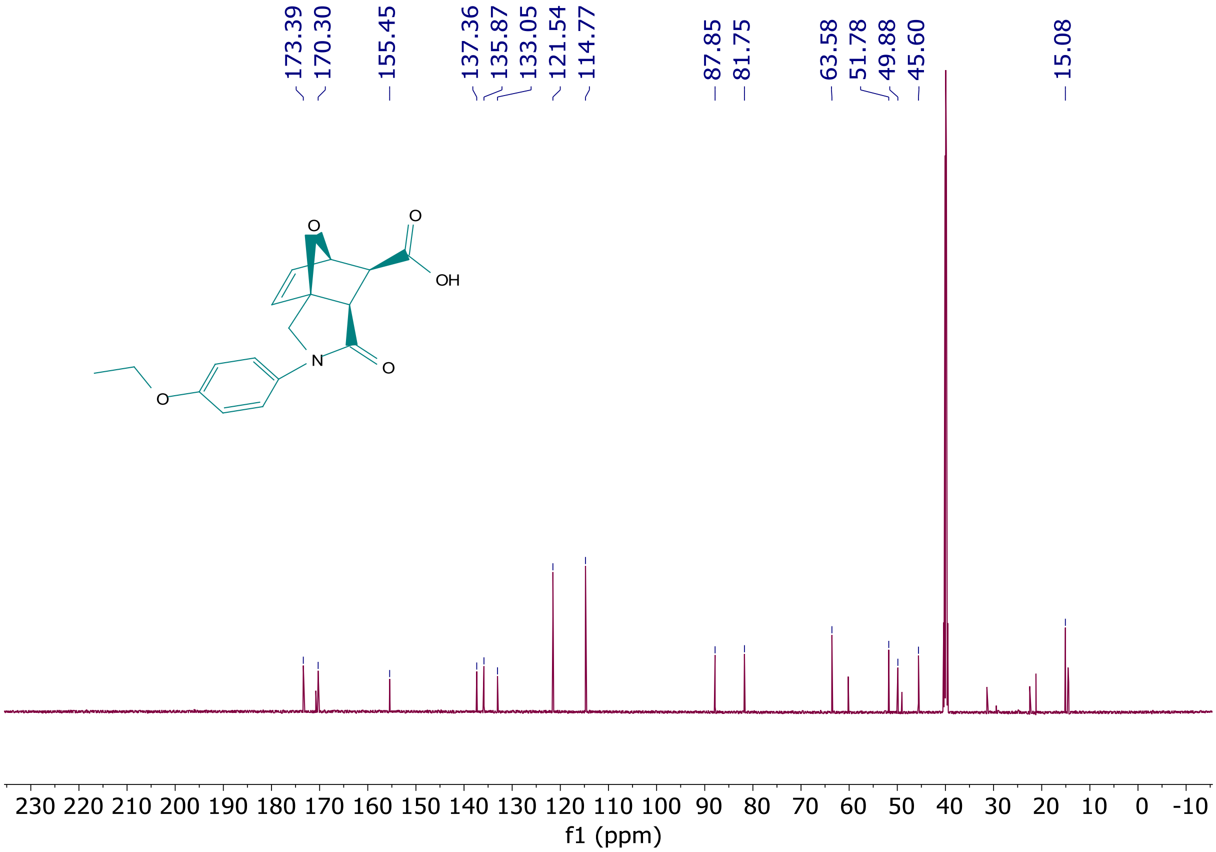

^{13}C NMR spectrum of compound **2m**

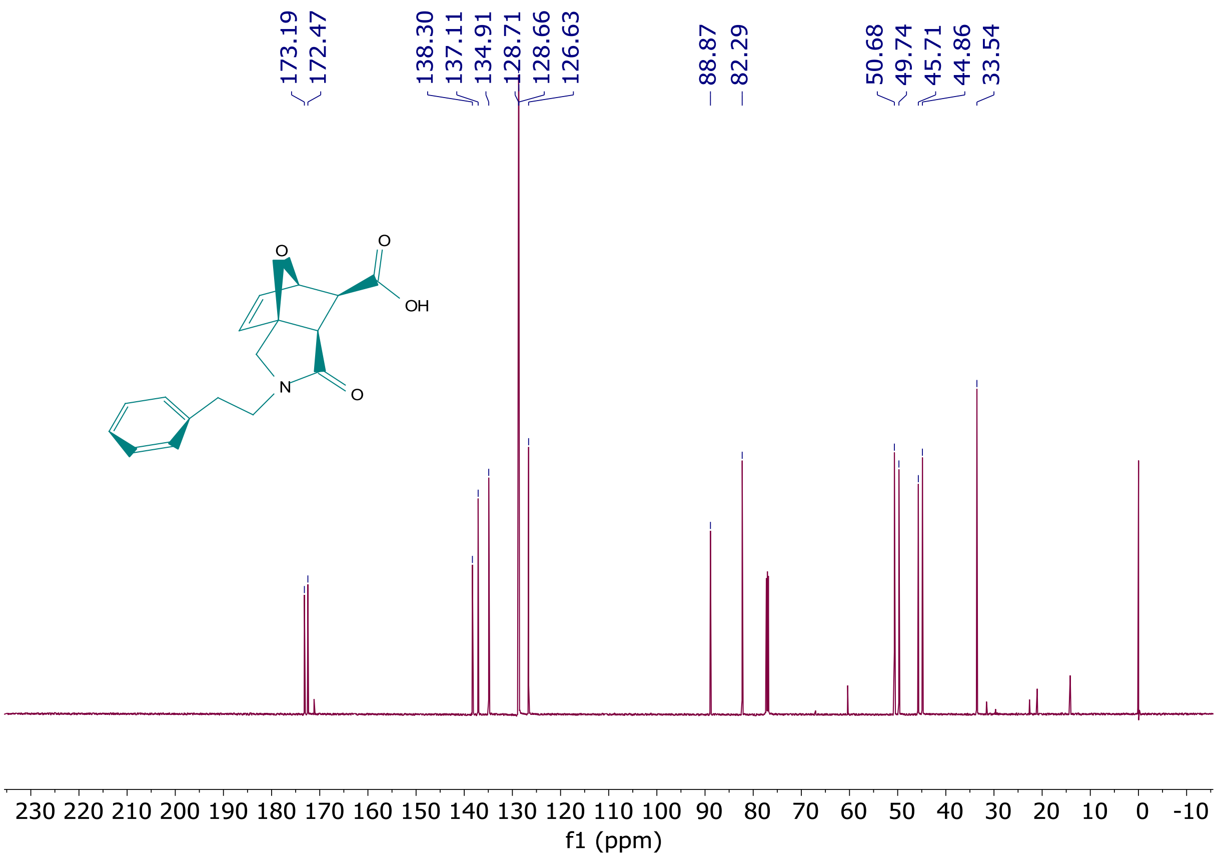

^{13}C NMR spectrum of compound **2n**

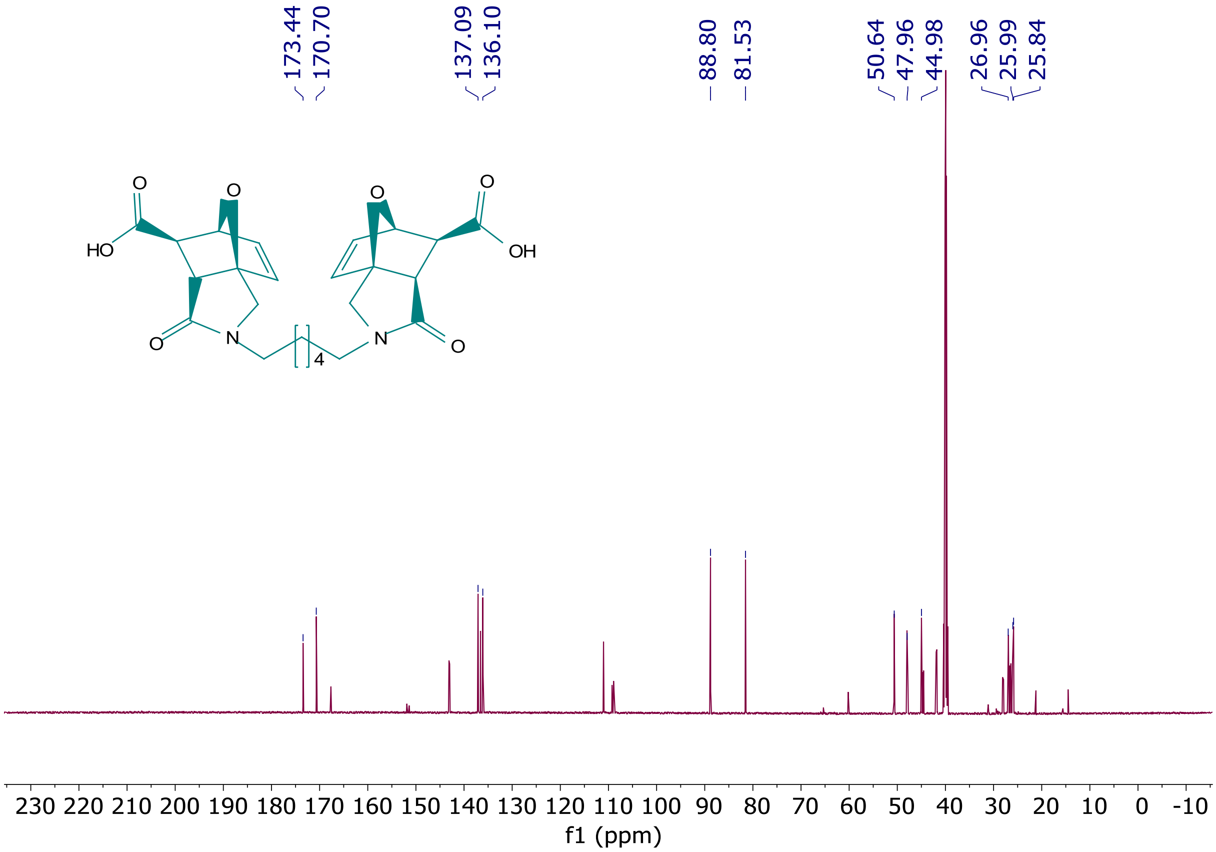

^{13}C NMR spectrum of compound **2o**

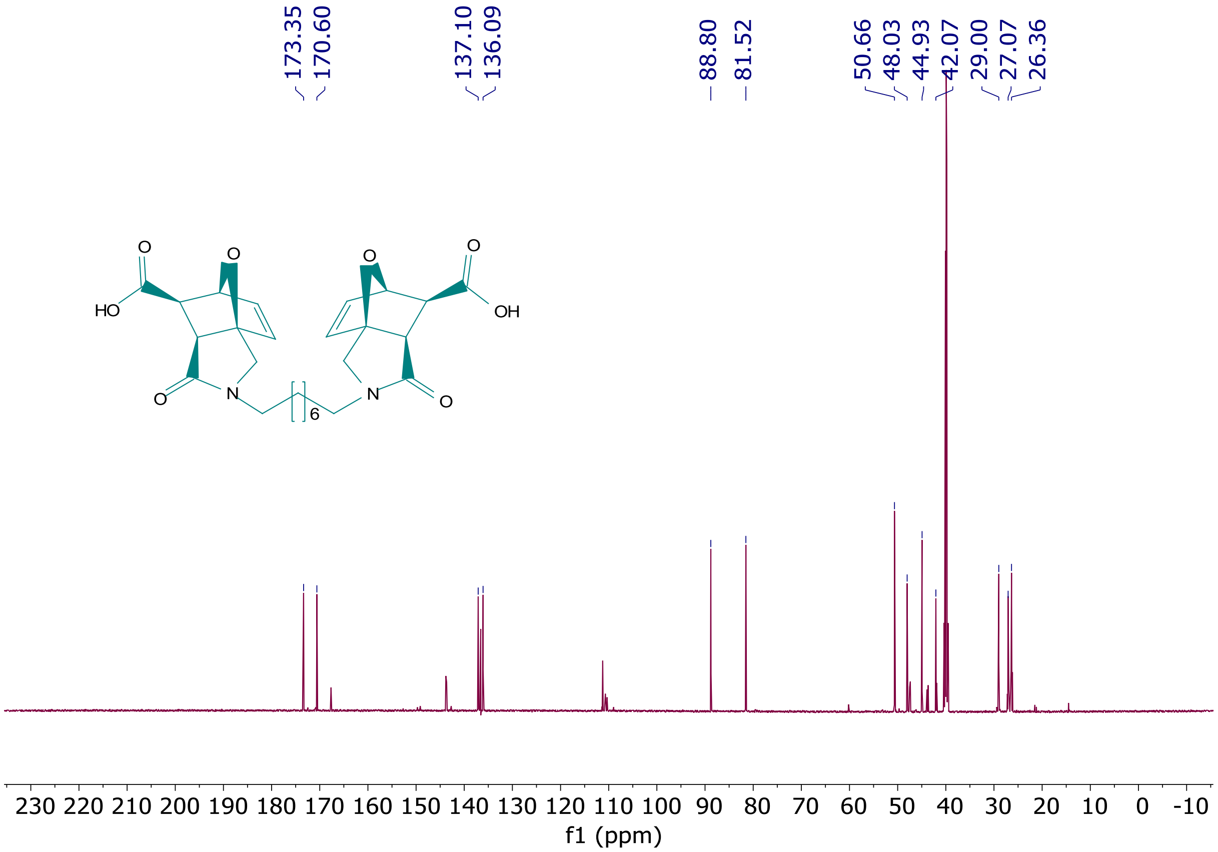

^{13}C NMR spectrum of compound **2p**

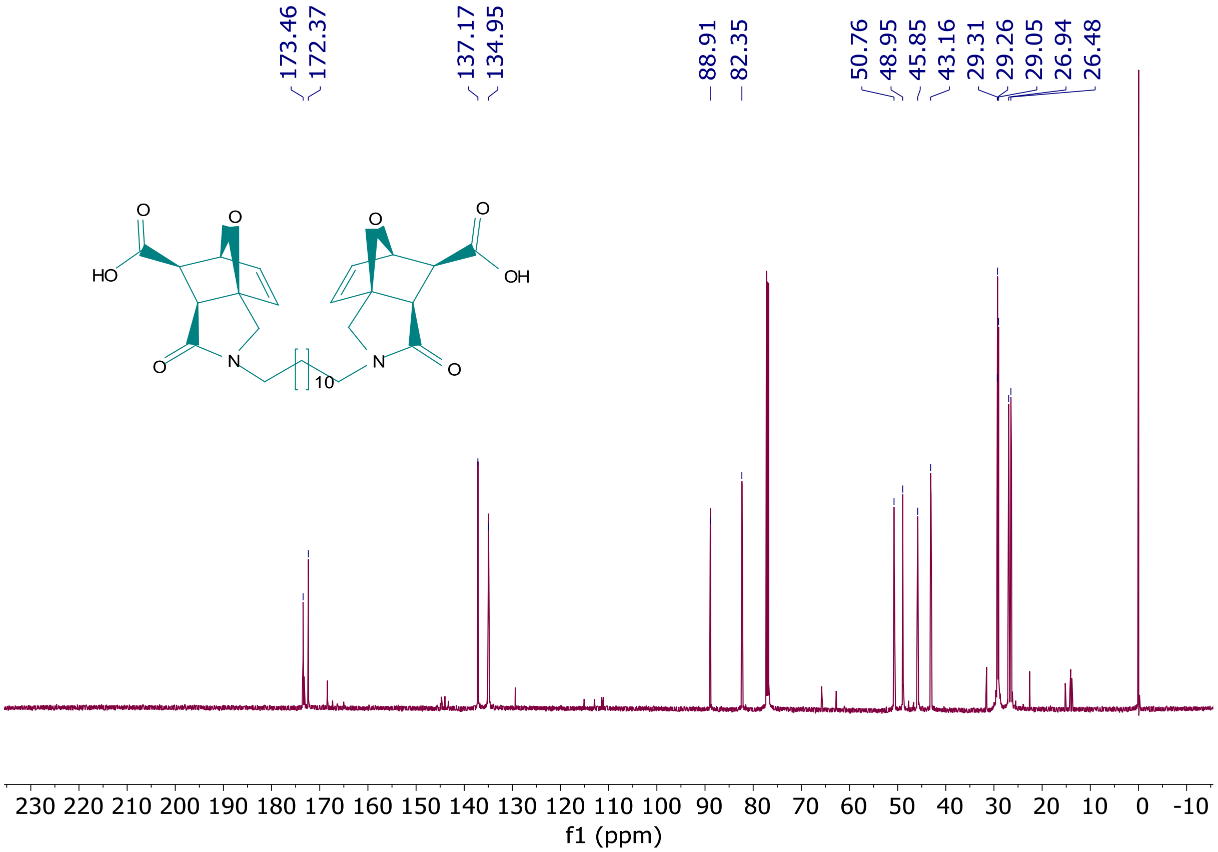

Endo reactant

0 1

C	-0.68105319	-0.42057247	-4.11655978
C	0.67717376	-0.61758367	-3.41798477
C	2.20217097	-0.68078790	-0.38879074
C	1.03385670	1.28062232	-0.51828232
C	1.15126655	0.25597251	0.26261424
C	2.00261092	0.95440145	-1.61502520
C	0.72519654	-1.78751769	-2.40935384
O	3.15491965	0.23470299	-0.94739991
O	-1.05028924	0.73221371	-4.46057830
O	-1.52266159	-1.54759791	-4.37436038
C	2.23788731	-1.94945660	0.28603198
C	-0.14091623	-2.09482392	-1.11056701
N	0.80701641	-2.90980363	0.11435415
O	-1.39120403	-2.01085108	-1.22567187
C	-0.18900290	-2.77756389	1.70676446
C	-0.16008257	-3.91513651	2.74441710
C	-0.10595988	-6.05373890	4.69522228
C	0.94594425	-3.95267374	3.81538034
C	-1.10798220	-4.88319819	2.71510692
C	-1.07921688	-6.02073575	3.75275397
C	0.97125142	-4.95369454	4.72854950
H	1.41933988	-0.77081073	-4.17337926
H	0.39064704	2.12997472	-0.41948176
H	0.60640214	0.07592032	1.16572718
H	2.27442299	1.83710026	-2.15529099
H	1.10095772	-2.61627141	-2.97226298
H	-2.44177875	-1.27081862	-4.35853577

H	2.31968236	-1.42768837	1.21660884
H	3.10313300	-2.56825575	0.17057691
H	-1.20041544	-2.58799675	1.41350027
H	0.22965087	-1.89173360	2.13685694
H	-0.08600498	-6.84414607	5.41621417
H	1.69443971	-3.18836860	3.83851036
H	-1.87639970	-4.85716564	1.97096744
H	-1.82768047	-6.78506326	3.72961340
H	1.73966316	-4.97970448	5.47270480

Endo TS

0 1

C	2.56949297	-0.03606042	0.89292158
C	1.35625953	0.09799597	1.66514517
C	1.95536874	0.94378641	4.01274227
C	3.10498688	-0.97728403	3.55171276
C	3.28364420	0.37019541	3.79190636
C	1.71067887	-1.23145297	3.69421548
C	1.11973698	1.22946305	2.48168094
O	1.09494876	-0.19205110	4.34592178
O	3.63339071	0.55447160	0.96286120
O	2.46222354	-1.07275396	-0.02478465
C	1.76002894	2.27773734	4.69238106
C	1.62892495	2.64682886	2.27555887
N	1.79862964	3.26242858	3.56219574
O	1.74617124	3.26272424	1.24570507
C	2.71984396	4.41766875	3.68779130
C	4.16814728	4.00964918	3.83127399
C	6.85320193	3.25532162	4.09453193
C	4.86938450	4.30687862	5.00761662
C	4.81512036	3.33458672	2.78510728
C	6.15309863	2.95749328	2.92013701
C	6.21048664	3.93145142	5.13569059
H	0.53789030	-0.56938396	1.41223471
H	3.84736140	-1.69915042	3.25260102
H	4.19355453	0.94887153	3.72065187
H	1.11298832	-2.11471683	3.58174209
H	0.04796750	1.29225386	2.80299571
H	3.27919049	-1.16253901	-0.57814799

H	2.54012312	2.48626701	5.45534980
H	0.77056544	2.34957724	5.19848292
H	2.37923655	5.03963539	4.54572322
H	2.59267521	5.05102584	2.77044431
H	7.89713445	2.96457035	4.19487782
H	4.37872286	4.83689506	5.82121173
H	4.27571472	3.09713283	1.86110073
H	6.64941974	2.43187549	2.10395399
H	6.75468215	4.16886387	6.04812797

Endo product

0 1

C	-0.02653584	0.15567848	-3.26308240
C	1.18873004	-0.42703240	-2.51798048
C	1.58884181	-1.59877832	-0.56364500
C	0.72178144	0.47077928	-0.19907896
C	0.62582889	-0.73219852	0.28058230
C	1.75978767	0.30967281	-1.27701791
C	0.96282978	-1.82182629	-1.89921826
O	2.71558141	-0.73057451	-0.74925765
O	-0.21130984	1.40017132	-3.28890296
O	-0.94502655	-0.71742925	-3.92562122
C	1.59102231	-2.96353643	0.00459331
C	-0.31471734	-2.40743388	-1.34966937
N	0.09959290	-3.25401072	-0.14356407
O	-1.48290703	-2.20884179	-1.77330197
C	-0.70627668	-2.81486741	1.00475094
C	-0.27258936	-3.59012705	2.26272066
C	0.54274250	-5.04761496	4.62770395
C	0.84573606	-3.03309792	3.16309102
C	-0.87507089	-4.76254117	2.57740808
C	-0.44138316	-5.53780111	3.83537747
C	1.22737918	-3.71532515	4.27010574
H	2.00859800	-0.47227376	-3.20403422
H	0.18327329	1.35017930	0.08651617
H	-0.01679527	-1.06190410	1.07003048
H	2.19986557	1.25499319	-1.51701960
H	1.34428573	-2.51955020	-2.61516010
H	-1.82011206	-0.32268860	-3.92334695

H	1.94422903	-3.05829932	1.01016032
H	2.18933747	-3.60010546	-0.61322449
H	-1.74076586	-3.00453236	0.80787704
H	-0.55970104	-1.76689949	1.16342930
H	0.84406981	-5.58626873	5.50174856
H	1.32142691	-2.10741729	2.91462856
H	-1.65208861	-5.14956834	1.95182560
H	-0.91707085	-6.46348403	4.08383743
H	2.00439586	-3.32829723	4.89568904

Exo reactant

0 1

C	-1.17820105	-1.23217408	-4.05694251
C	-0.77913379	0.04024213	-3.28375272
C	1.52558979	1.28522061	-1.01440636
C	1.66418979	2.81962061	-2.65290636
C	1.63778979	2.74372061	-1.31310636
C	1.62133379	1.40915787	-3.17484728
C	-0.87328979	0.09397939	-1.75019364
O	2.34388979	0.75652061	-2.09840636
O	-1.03230463	-2.19415155	-3.32016370
O	-1.56647570	-1.40957828	-5.33206227
C	1.70179355	0.48812966	0.50614496
C	-0.77057348	-1.20326561	-1.01991228
N	0.62865641	-1.21745628	0.42594412
O	-1.56497348	-2.09536561	-1.27051228
C	0.75645641	-2.26515628	1.41834412
C	0.17185641	-1.85305628	2.74644412
C	-0.93054359	-1.05155628	5.19764412
C	-1.21924359	-1.78695628	2.91514412
C	1.00355641	-1.50715628	3.82084412
C	0.45335641	-1.11025628	5.04104412
C	-1.76674359	-1.38935628	4.13534412
H	-1.37897919	0.79844458	-4.19903091
H	1.68678979	3.71102061	-3.26050636
H	1.62358979	3.56042061	-0.60980636
H	2.10703379	1.24225787	-4.13784728
H	-1.68328979	0.71487939	-1.35049364
H	-1.68611518	-2.37828911	-5.42474739

H	1.33919355	1.09142966	1.34384496
H	2.76039355	0.24252966	0.62884496
H	1.81955641	-2.51355628	1.51434412
H	0.24705641	-3.17025628	1.06714412
H	-1.35784359	-0.74415628	6.14844412
H	-1.88114359	-2.04755628	2.09044412
H	2.08525641	-1.54625628	3.71514412
H	1.10445641	-0.84755628	5.87094412
H	-2.84594359	-1.34465628	4.25574412

Exo TS

0 1

C	0.69460351	0.32751386	0.63243955
C	0.19369104	-0.16994905	1.92314545
C	2.00411275	-0.35500348	3.88841768
C	0.35042481	1.11917008	4.33629712
C	0.99998701	0.00468662	4.84987818
C	1.00321884	1.44586990	3.10649324
C	0.76698338	-1.26131406	2.58543085
O	2.22609855	0.77985224	3.06041605
O	1.67834133	0.08632787	-0.02368892
O	-0.19666040	1.28641230	0.15545271
C	3.15945274	-1.29719849	3.90256113
C	1.80561692	-2.18884907	2.01127252
N	3.02418311	-2.16419141	2.70275480
O	1.60959075	-2.96978918	1.09897063
C	4.12668433	-3.09374328	2.37085417
C	4.08568933	-4.32318320	3.24882316
C	3.98792417	-6.61371732	4.85476617
C	3.08465162	-5.28463026	3.04158381
C	5.03519379	-4.50936999	4.26163605
C	4.98502231	-5.65596804	5.06118542
C	3.03787994	-6.42611938	3.84414998
H	-0.84655713	0.09895536	2.13926755
H	-0.51538646	1.61749937	4.73341690
H	0.75669896	-0.55142970	5.73651279
H	0.93519804	2.30373215	2.46534568
H	0.12463611	-1.80077392	3.30096726
H	0.06778335	1.63276953	-0.73512118

H	3.17601134	-1.91670416	4.83059866
H	4.12699786	-0.73900668	3.85320944
H	5.09907992	-2.55980965	2.45007361
H	4.01704473	-3.38875756	1.29286195
H	3.95159120	-7.50581591	5.47684699
H	2.34605596	-5.13194412	2.24921692
H	5.81647094	-3.77079525	4.42574100
H	5.72637804	-5.80276583	5.84459581
H	2.26225513	-7.17289357	3.68050961

Exo product

0 1

C	-1.41447282	-0.57320259	-2.99072490
C	-0.47252564	0.50691805	-2.42709831
C	1.35533894	0.42819564	-0.99087127
C	1.47756984	2.02617597	-2.52624057
C	1.70538188	1.88524403	-1.22076937
C	0.97418548	0.63584588	-3.00098750
C	-0.10135286	0.16025758	-0.97572353
O	1.78278102	-0.25570216	-2.22575231
O	-1.39707297	-1.73615905	-2.51030834
O	-2.30891061	-0.25463031	-4.06002034
C	1.84042425	-0.25398406	0.27562569
C	-0.03660817	-1.33045956	-0.59755066
N	1.28680151	-1.61569116	0.04294328
O	-0.94540323	-2.17637293	-0.80271564
C	1.20734103	-2.43232988	1.26265113
C	0.32212308	-1.71883951	2.30136219
C	-1.34208676	-0.37747714	4.25413858
C	-1.19432562	-1.98519628	2.33357074
C	0.87760615	-0.85657403	3.18708440
C	-0.00761188	-0.14308328	4.22579515
C	-1.97331746	-1.35732455	3.24763630
H	-0.93768854	1.45942077	-2.57291339
H	1.58929662	2.91358394	-3.11354307
H	2.02988756	2.61874709	-0.51255378
H	1.03483534	0.51468220	-4.06237383
H	-0.73524303	0.64995376	-0.26630061
H	-1.87572888	-0.42209728	-4.90020389

H	1.40592422	0.21602683	1.13306343
H	2.90273979	-0.25737893	0.40358727
H	2.18938747	-2.56923580	1.66482219
H	0.78251005	-3.38558488	1.02659278
H	-1.95714084	0.11825993	4.97584021
H	-1.63290750	-2.66600193	1.63424991
H	1.93124287	-0.67150940	3.16470697
H	0.43097017	0.53772147	4.92511673
H	-3.02695344	-1.54239302	3.27001700
